# HLA-B*57:01-dependent intracellular stress in keratinocytes triggers dermal hypersensitivity reactions to abacavir

**DOI:** 10.1093/pnasnexus/pgae140

**Published:** 2024-04-02

**Authors:** Akira Kazaoka, Sota Fujimori, Yushiro Yamada, Tomohiro Shirayanagi, Yuying Gao, Saki Kuwahara, Naoki Sakamoto, Takeshi Susukida, Shigeki Aoki, Kousei Ito

**Affiliations:** Laboratory of Biopharmaceutics, Graduate School of Pharmaceutical Sciences, Chiba University, Chiba 260-8675, Japan; Laboratory of Biopharmaceutics, Graduate School of Pharmaceutical Sciences, Chiba University, Chiba 260-8675, Japan; Laboratory of Biopharmaceutics, Graduate School of Pharmaceutical Sciences, Chiba University, Chiba 260-8675, Japan; Laboratory of Biopharmaceutics, Graduate School of Pharmaceutical Sciences, Chiba University, Chiba 260-8675, Japan; Laboratory of Biopharmaceutics, Graduate School of Pharmaceutical Sciences, Chiba University, Chiba 260-8675, Japan; Laboratory of Biopharmaceutics, Graduate School of Pharmaceutical Sciences, Chiba University, Chiba 260-8675, Japan; Laboratory of Biopharmaceutics, Graduate School of Pharmaceutical Sciences, Chiba University, Chiba 260-8675, Japan; Laboratory of Biopharmaceutics, Graduate School of Pharmaceutical Sciences, Chiba University, Chiba 260-8675, Japan; Laboratory of Cancer Biology and Immunology, Section of Host Defences, Institute of Natural Medicine, University of Toyama, Toyama 930-0194, Japan; Laboratory of Biopharmaceutics, Graduate School of Pharmaceutical Sciences, Chiba University, Chiba 260-8675, Japan; Laboratory of Biopharmaceutics, Graduate School of Pharmaceutical Sciences, Chiba University, Chiba 260-8675, Japan

**Keywords:** HLA, endoplasmic reticulum stress, abacavir, drug eruption, keratinocytes

## Abstract

Specific human leukocyte antigen (HLA) polymorphisms combined with certain drug administration strongly correlate with skin eruption. Abacavir hypersensitivity (AHS), which is strongly associated with HLA-B*57:01, is one of the most representative examples. Conventionally, HLA transmits immunological signals via interactions with T cell receptors on the cell surface. This study focused on HLA-mediated intracellular reactions in keratinocytes that might determine the onset of skin immunotoxicity by drug treatments. Abacavir exposure resulted in keratinocytes expressing HLA-B*57:01 exhibiting endoplasmic reticulum (ER) stress responses, such as immediate calcium release into the cytosol and enhanced HSP70 expression. In contrast, keratinocytes expressing HLA-B*57:03 (closely related to HLA-B*57:01) did not show these changes. This indicated that HLA-B*57:01 has a specific intracellular response to abacavir in keratinocytes in the absence of lymphocytes. Furthermore, abacavir exposure in HLA-B*57:01-expressing keratinocytes elevated the expression of cytokines/chemokines such as interferon-γ, interleukin-1β, and CCL27, and induced T lymphoblast migration. These effects were suppressed by ER stress relief using 4-phenylbutyrate (4-PB). HLA-B*57:01-transgenic mice also exhibited ER stress in epidermal areas following abacavir administration, and abacavir-induced skin toxicity was attenuated by the administration of 4-PB. Moreover, abacavir bound to HLA-B*57:01 within cells and its exposure led to HLA-B*57:01 protein aggregation and interaction with molecular chaperones in the ER of keratinocytes. Our results underscore the importance of HLA-mediated intracellular stress responses in understanding the onset of HLA-B*57:01-mediated AHS. We provide the possibility that the intracellular behavior of HLA is crucial for determining the onset of drug eruptions.

**Significance Statement**

Human leukocyte antigen (HLA) presents intracellular antigens to T cells on the cell surface and generates immunological signals. The development of HLA polymorphism-dependent drug eruptions, such as abacavir hypersensitivity involving HLA-B*57:01, has conventionally been understood to trigger drug effects on antigen presentation. While this conventional theory explains the mechanism of immune activation, it does not fully account for the development of skin eruptions. Our study revealed HLA polymorphism-dependent endoplasmic reticulum stress within keratinocytes in response to abacavir, followed by subsequent immunological responses. Furthermore, we found that HLA binds to abacavir intracellularly and forms intracellular aggregates upon abacavir exposure. Our findings suggest that elucidating the intracellular behavior of HLA can enhance our understanding of the pathogenesis of drug eruptions.

## Introduction

Individual differences in adverse drug reactions (ADRs) arise due to various genes and environmental factors. Recent genome-wide association studies have emphasized that the onset of many ADRs is strongly associated with specific human leukocyte antigen (HLA) alleles (1, 2). Severe cutaneous adverse reactions (SCARs) and drug-induced liver injury are major HLA-mediated ADRs (1, 2). Abacavir hypersensitivity (AHS) with skin eruption is associated with the HLA-B*57:01 allele (3–6). Other representative combinations include HLA-B*58:01 linked to allopurinol-induced Stevens–Johnson syndrome (SJS) and toxic epidermal necrolysis (TEN) (7–9), HLA-B*13:01 associated with dapsone-induced SCARs (10), HLA-B*15:02 related to carbamazepine-induced SJS/TEN (11, 12), and HLA-A*31:01 linked to carbamazepine-induced drug reaction with eosinophilia and systemic symptoms (13). Elucidating how these HLAs influence the onset of SCARs can prevent adverse outcomes.

HLA is classified into HLA class I and class II. Specific polymorphisms of HLA class I are associated with SCARs (1, 2, 14, 15). HLA class I is composed of an HLA heavy chain and β_2_-microglobulin (β_2_m). Antigenic peptides are loaded onto HLA within the endoplasmic reticulum (ER). On the cell surface, peptide-loaded HLA presents antigens to CD8^+^ T cell receptors (TCRs), thereby enabling cytotoxic T cell activity against non-self-antigens. The pathogenesis of SCARs has traditionally been explained by focusing on the presentation of peptide-loaded HLA to TCRs. Conventional hypotheses include: (i) presenting covalently bound drug-self peptides during HLA antigen presentation (the hapten/prohapten model), (ii) direct non-covalent binding of drugs to immune receptors such as TCRs and HLA (the pharmacological interaction with immune receptors model), and (iii) presenting altered self-peptide-loaded HLA due to non-covalent drug binding (the altered peptide repertoire model) (1). While experiments involving lymphocytes or peripheral blood mononuclear cells (PBMCs) have supported these hypotheses (6, 16, 17) (18–23), they alone do not fully account for the pathogenesis of SCARs. HLA class I is expressed in all somatic cells and can present peptides. According to conventional hypotheses, ADRs involving inflammation should not be limited to skin tissue alone but could manifest in other tissues as the primary symptom. However, SCARs are more likely to occur than liver injury, kidney injury, and blood disorders (1, 2, 14), suggesting that conventional hypotheses alone are insufficient to explain the mechanism of HLA-mediated SCARs.

The pathogenesis of HLA class I-mediated SCARs has been elucidated through studies of AHS, which is strongly associated with HLA-B*57:01 (3–6). The pathogenesis of AHS has been hypothesized to occur when abacavir binds to the peptide-binding groove of HLA-B*57:01 during peptide loading in ER. The altered peptide repertoire on the cell surface is then recognized as foreign by CD8^+^ T cells (20–22). Additional focus has been given to molecules other than HLA that are required for AHS development. Specifically, genetic variants of heat shock protein (HSP) 70-Hom and ER aminopeptidase 1, which are pivotal in protein folding and peptide loading upon HLA, have been reported to be associated with AHS (24–26). Therefore, the role of HLA in intracellular processes, such as the intracellular assembly of HLA, merits consideration. Indeed, our previous studies have shown that exposure to abacavir in HLA-B*57:01-expressing cells leads to the formation of abnormal HLA complexes. These complexes fail to assume heterotrimeric structures with β_2_m and peptides (27), suggesting that abacavir affects the intracellular assembly of HLA complexes. Consequently, focusing on the intracellular molecular behavior of HLA-B*57:01 is important for understanding the mechanism of AHS and, ultimately, the pathogenesis underlying HLA-mediated SCARs.

A diverse network of immune cells resides in skin tissue and plays a crucial role in maintaining tissue homeostasis (28–30). Among these, keratinocytes, the predominant cell type in the epidermis, serve as the primary innate immune cells of the skin (31). Specifically, keratinocytes are characterized by the secretion of interleukin (IL)-1 when stimulated by external stressors such as haptens and injuries, as well as the release of various cytokines and chemokines through IL-1 stimulation (31–34). Additionally, keratinocytes possess toll-like receptors; upon pathogen recognition, these receptors activate toll-like receptor signaling pathways, leading to the production of various cytokines and chemokines such as interferon (IFN)-γ and C-C motif chemokine ligand 27 (32, 35). These cytokines and chemokines induce the activation, clustering, and migration of T cells, macrophages, and dendritic cells in skin tissues, leading to skin inflammation (31, 32). Keratinocytes are highly significant when considering the pathogenesis of HLA-mediated SCARs.

We have previously generated chimeric HLA-B*57:01 transgenic mice (B*57:01-Tg) and demonstrated HLA-B*57:01-dependent skin eruption upon abacavir administration in these mice (36–39). In this study, we investigated the response of keratinocytes isolated from B*57:01-Tg to abacavir exposure to clarify the pathogenesis of abacavir-induced immunotoxic reactions in the skin.

## Results

### HLA-B*57:01-expressing keratinocytes were immunologically activated by abacavir

Immune-modulating factors secreted by keratinocytes play a major role in cutaneous inflammatory responses (31, 32). First, we quantified the expression of mRNAs encoding proinflammatory cytokines in abacavir-treated keratinocytes from HLA-Tg. Keratinocytes derived from B*57:01-Tg (B*57:01-KCs), their littermates (LM; LM-KCs), and B*57:03-Tg (a negative control mice possessing a closely related allele to HLA-B*57:01; B*57:03-KCs) were exposed to abacavir for 12 h (Fig. S1a).

**Fig. 1:**
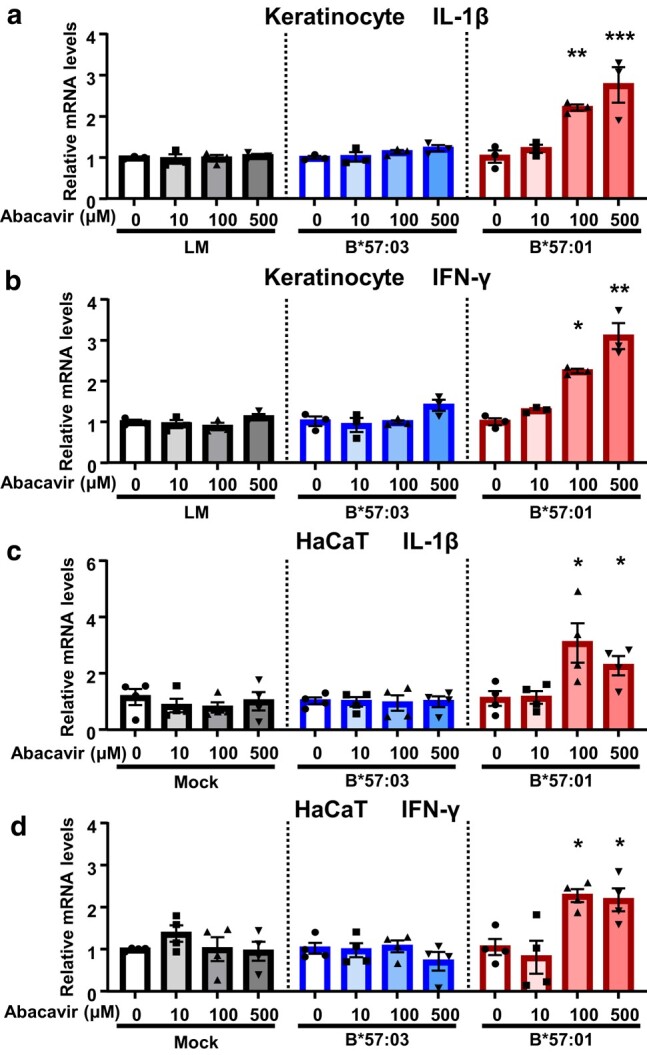
. Immunological activation of HLA-B*57:01-expressing keratinocytes by abacavir. Panels a, b show mRNA expression levels of IL-1β a) and IFN-γ b) relative to GAPDH in keratinocytes from B*57:01-Tg, their LM, and B*57:03-Tg. Panels c and d display mRNA expression levels of IL-1β c) and IFN-γ d) relative to GAPDH in B*57:01-, B*57:03-, or mock mRNA-transfected HaCaT cells. Both keratinocytes and HaCaT cells were incubated in the presence or absence of abacavir for 12 h. mRNA levels are presented relative to the mean value in keratinocytes or HaCaT cells incubated without abacavir. Data are expressed as the mean ± SEM (*n* = 3/group). Significant differences (* *P* < 0.05, ** *P* < 0.01, *** *P* < 0.001) were observed between keratinocytes incubated in the presence and absence of abacavir, as determined by one-way ANOVA followed by Dunnett's multiple comparisons tests.

Significant dose-dependent increases in the mRNA expression levels of IL-1β and IFN-γ were observed in B*57:01-KCs due to abacavir exposure. In contrast, no significant elevation in the levels of these mRNAs was observed in LM-KCs or B*57:03-KCs (Fig. 1a and b). Similar patterns of mRNA expression changes were observed in HLA-transfected HaCaT cells, spontaneously immortalized human keratinocytes (Figs. 1c, d, and S1b). Protein levels of IFN-γ and IL-1β in the culture supernatant of B*57:01-KC were below the detection limit (15.6 pg/mL) with or without abacavir exposure (100 μM, 12 h). Exposure to abacavir (up to 1,000 μM) for 24 h did not show remarkable cytotoxicity in keratinocytes (Fig. S2). These results suggest that HLA-B*57:01-expressing keratinocytes have the potential to respond to abacavir and to induce several proinflammatory cytokine production, which did not result from cytotoxic reactions.

However, in HLA-B*57:01-transfected HeLa cells and hepatocytes from B*57:01-Tg, no significant difference in the mRNA expression levels of IL-1β and IFN-γ was observed with abacavir exposure (Fig. S3).

### Comprehensive mRNA expression analysis revealed HLA-B*57:01-dependent responses to abacavir in keratinocytes

**Fig. 2.**
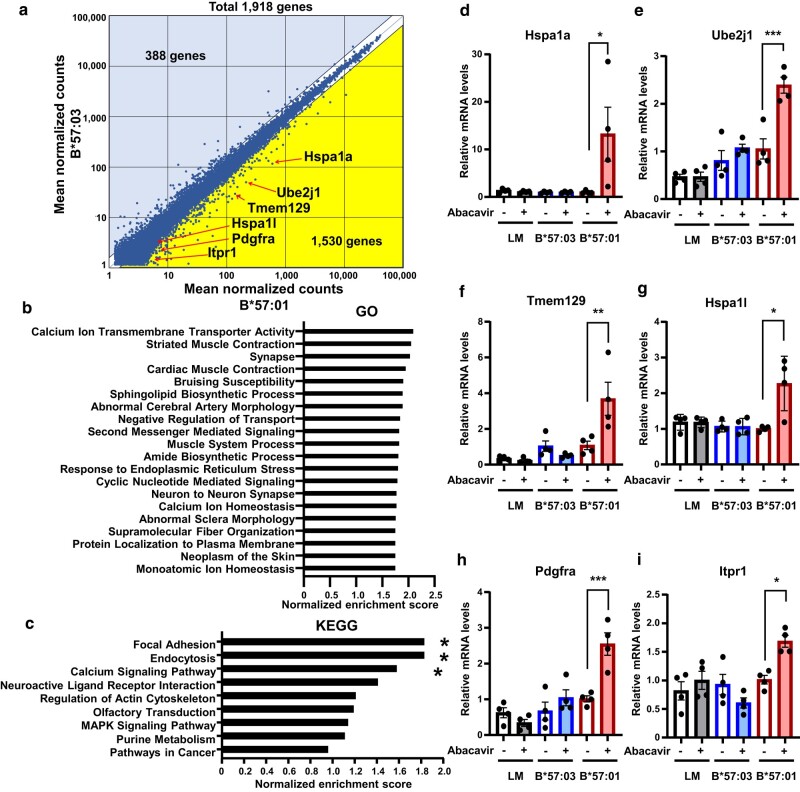
Comprehensive mRNA expression analysis uncovers HLA polymorphism-dependent responses to abacavir in keratinocytes. a) Scatter plot showing 3D-Gene DNA microarray hybridization intensities for abacavir-exposed keratinocytes of B*57:01-Tg compared to keratinocytes of B*57:03-Tg. b) Positively enriched GO gene sets in abacavir-exposed keratinocytes of B*57:01-Tg relative to B*57:03-Tg. In this panel, gene sets with significant differences (nominal [NOM] *P* < 0.01). c) Positively enriched KEGG gene sets in abacavir-exposed keratinocytes of B*57:01-Tg relative to B*57:03-Tg. KEGG pathway gene sets are listed according to their normalized enrichment score. Significant differences (* NOM *P* < 0.05) were observed between abacavir-exposed keratinocytes of B*57:01-Tg and B*57:03-Tg. d–i) mRNA expression levels of Hspa1a d), Ube2j1 e), Tmem129 f), Hspa1 l g), Pdgfra h), and Itpr1 i) relative to GAPDH in keratinocytes from B*57:01-Tg, their LM, and B*57:03-Tg. Keratinocytes were incubated with (+) or without (−) 100 μM abacavir for 12 h. mRNA levels are presented relative to the mean value in keratinocytes from B*57:01-Tg without abacavir exposure. Data are expressed as the mean ± SEM (*n* = 4/group). Significant differences (* *P* < 0.05, ** *P* < 0.01, *** *P* < 0.001) were observed compared with another group (one-way ANOVA, followed by Bonferroni’s multiple comparisons tests; d–i).

We conducted a comprehensive analysis of gene expression in abacavir-exposed B*57:01-KCs using DNA microarray analysis and compared the results with those of abacavir-exposed B*57:03-KCs (Figs. 2a–c and Fig. S4; https://www.ncbi.nlm.nih.gov/geo/; accession No. GSE110394). Gene Ontology (GO) analysis and Kyoto Encyclopedia of Genes and Genomes (KEGG) pathway analyses revealed an increase in several gene sets which included calcium ion transmembrane transporter activity, response to ER stress, and calcium signaling pathway, suggesting the occurrence of ER stress (Figs. 2b, c, and S4a–c; Table S1) (40). Consequently, we confirmed that abacavir exposure significantly increased the mRNA expression of representative genes (Hspa1a, Ube2j1, Tmem129, Hsp1al, Pdgfra, Itpr1) in B*57:01-KCs (Fig. 2d–i). In contrast, no significant changes were observed in LM-KCs or B*57:03-KCs (Fig. 2d–i).

These results suggest that HLA-B*57:01-expressing keratinocytes can exhibit various intracellular responses, including ER stress response, upon exposure to abacavir.

### ER stress responses in HLA-B*57:01-expressing keratinocytes were induced by abacavir

**Fig. 3.**
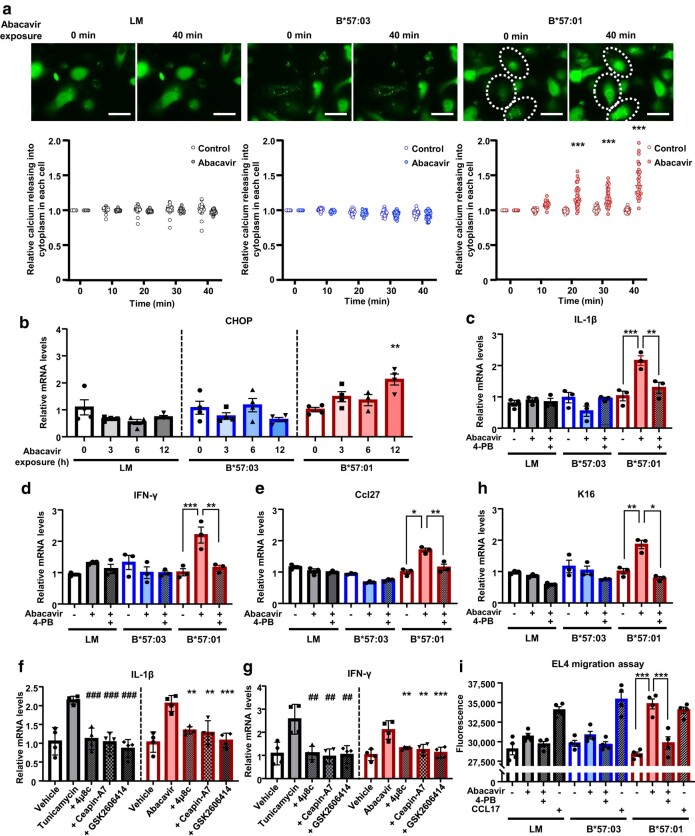
Abacavir induces ER stress responses in HLA-B*57:01-expressing keratinocytes. a) Chronological changes in calcium release from the ER to the cytoplasm in keratinocytes of B*57:01-Tg, their LM, and B*57:03-Tg with (+) or without (−) 100 μM abacavir exposure. Calcium release was observed and measured using Cal-520 AM. Each scale bar represents 50 μm. Fluorescence levels are presented relative to 0 min after the start of incubation with fresh medium, with or without abacavir, for each cell. Data are expressed as the mean ± SEM (*n* = 30/group). b) mRNA expression levels of CHOP relative to GAPDH in keratinocytes from B*57:01-Tg, LM, and B*57:03-Tg. Keratinocytes were incubated with 100 μM abacavir for 0, 3, 6, and 12 h. mRNA levels are presented relative to the mean value in keratinocytes with abacavir exposure for 0 h. Data are expressed as the mean ± SEM (*n* = 3–4/group). c, d, e, h) mRNA expression levels of IL-1β c), IFN-γ d), Ccl27 e), and K16 h) relative to GAPDH in keratinocytes of B*57:01-Tg, LM, and B*57:03-Tg. Following 4-PB pretreatment, keratinocytes were incubated with (+) or without (−) 100 μM abacavir for 12 h. mRNA levels are presented relative to the mean value in keratinocytes from B*57:01-Tg without abacavir exposure. Data are expressed as the mean ± SEM (*n* = 3/group). f, g) mRNA expression levels of IL-1β f) and IFN-γ g) relative to GAPDH in keratinocytes of B*57:01-Tg. Following 4μ8c (IRE1 pathway inhibitor), ceapin-A7 (ATF6 pathway inhibitor), or GSK2606414 (PERK pathway inhibitor) pretreatment, keratinocytes were incubated with 100 μM abacavir, 1.0 μg/mL tunicamycin, or their vehicle for 12 h. mRNA levels are presented relative to the mean value in keratinocytes from B*57:01-Tg incubated with vehicle. Data are expressed as the mean ± SEM (*n* = 4/group). i) EL4 migration ability during coculture with keratinocytes from B*57:01-Tg, LM, and B*57:03-Tg. After 4-PB pretreatment, keratinocytes in the lower chamber were incubated with (+) or without (−) 100 μM abacavir for 48 h, while DiI-dyed EL4 cells were cultured in the upper chamber. DiI fluorescence in the lower chamber was measured to assess EL4 migration. Data are expressed as the mean ± SEM (*n* = 4/group). Significant differences (*** *P* < 0.001) were also found between keratinocytes with and without abacavir exposure (Student's t test; a). Significant differences (* *P* < 0.05, ** *P* < 0.01, *** *P* < 0.001) were observed compared with another group (one-way ANOVA, followed by Bonferroni’s multiple comparisons tests; c–e, h, i). Significant differences (## *P* < 0.01, ### *P* < 0.001 vs. tunicamycin exposure; ** *P* < 0.01, *** *P* < 0.001 vs. abacavir exposure) were observed compared with another group (one-way ANOVA, followed by Bonferroni’s multiple comparisons tests; f, g).

We speculated that keratinocytes can induce ER stress response in an HLA-B*57:01-dependent manner under the influence of abacavir (Fig. 2). A follow-up observation of calcium release after abacavir exposure showed enhanced fluorescence of the calcium fluorescent probe in abacavir-exposed B*57:01-KCs (Fig. 3a, Movie S1). In addition to calcium release, the ER stress response induces unfolded protein response (UPR) through activation of ER stress sensors: inositol-requiring enzyme 1, activating transcription factor 6, and protein kinase R-like endoplasmic reticulum kinase (PERK). UPR induces transcription of mRNAs such as CCAAT-enhancer-binding protein homologous protein (CHOP) (41, 42). Significant time-dependent increases in the mRNA expression levels of CHOP were found in B*57:01-KCs by abacavir exposure (Fig. 3b). These phenomena were not observed in LM-KCs or B*57:03-KCs (Fig. 3a and b). These results imply that keratinocytes induce ER stress due to abacavir exposure in an HLA-B*57:01-dependent manner.

ER stress relief using pretreatment with the chemical chaperone 4-phenylbutyrate (4-PB) (43, 44) suppressed the increase in mRNA levels of IL-1β, IFN-γ, and CCL27 in abacavir-exposed B*57:01-KCs and B*57:01 mRNA-transfected HaCaT cells (Figs. 3c–e and S5a–c). Exposure to inhibitors of each UPR pathway (IRE1, ATF6, and PERK pathways) (45) also suppressed the increase in mRNA levels of IL-1β and IFN-γ induced in abacavir-exposed B*57:01-KCs (Fig. 3f and g). Increased cytokine expression correlates with elevated cytokeratin 16 (K16) expression in keratinocytes (46). We have previously reported that oral administration of abacavir to B*57:01-Tg increased K16 expression in epidermal cells (47). The expression level of K16 also increased in B*57:01-KCs and B*57:01 mRNA-transfected HaCaT cells exposed to abacavir, and this increase was suppressed by pretreatment with 4-PB (Figs. 3h and S5d). These results indicate that abacavir could alter the immunological properties of keratinocytes through HLA-B*57:01-dependent ER stress.

Keratinocytes promote T cell infiltration by secreting various cytokines and chemokines (30). Abacavir-exposed B*57:01-KCs induced the migration of the T lymphoblast cell line EL4, which was suppressed by 4-PB treatment (Fig. 3i). These findings suggest that cytokines and chemokines released from HLA-B*57:01-expressing keratinocytes due to abacavir exposure can enhance the migratory potential of immune cells.

### ER stress relief attenuated Abacavir-induced skin eruption in B*57:01-Tg

**Fig. 4.**
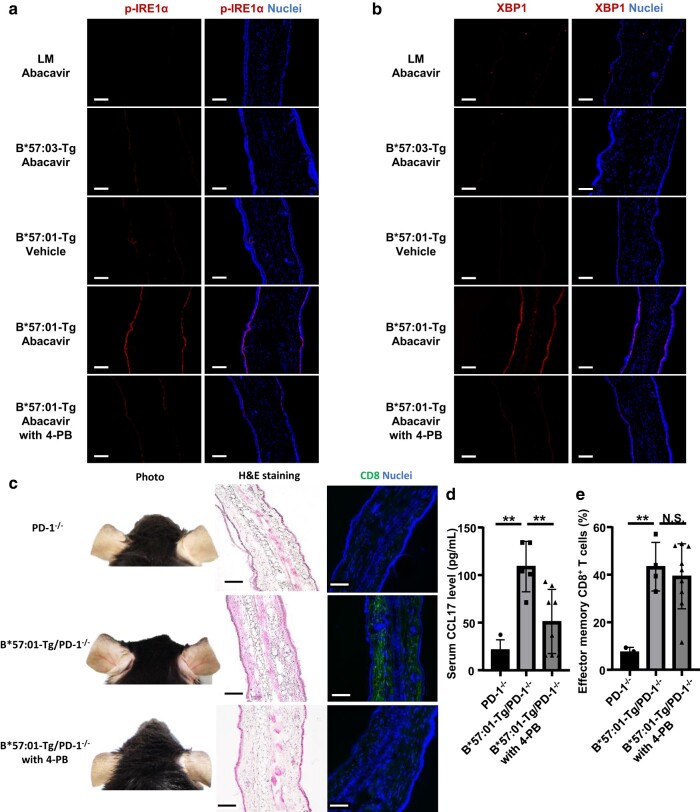
ER stress relief attenuates abacavir-induced skin eruption in B*57:01-Tg. Panels a and b show representative images of auricular sections stained with antiphosphorylated IRE1α (*p*-IRE1α; a) or XBP1 b) antibody, along with Hoechst 33342 for nuclear staining in B*57:01-Tg, their LM, and B*57:03-Tg. Mice were treated with 20 mg/body of abacavir p.o. and either received or did not receive 4-PB treatment 3 h before auricular tissue removal. Images are representative of three independent experiments (Fig. S7a and b). Each scale bar represents 100 μm. Panel c shows representative images of auricular sections stained with either H&E or anti-CD8 antibody and Hoechst 33342 in CD4^+^ T cell-depleted, PD-1-deficient B*57:01-Tg (B*57:01-Tg/PD-1^−/−^) and their LM (PD-1^−/−^). Mice were treated with 20 mg/body/day of abacavir p.o. for 5 days, with or without 4-PB treatment. Images are representative of three to five independent experiments (Fig. S8). Each scale bar represents 100 μm. Panels d and e show serum CCL17 levels d) and percentages of effector memory T cells (CD44^high^CD62L^low^) among CD8^+^ T cells isolated from the auricular lymph nodes e) in mice treated with abacavir and 4-PB. CD4^+^ T cell-depleted B*57:01-Tg/PD-1^−/−^ or PD-1^−/−^ mice were treated with 20 mg/body/day of abacavir p.o. for 5 days, with or without 4-PB treatment. Serum levels below the limit of detection are plotted as 15.7 pg/mL d). Data are expressed as the mean ± SD (*n* = 3–9/group). Differences were observed (N.S. not significant, ** *P* < 0.01) when compared with another group (one-way ANOVA, followed by Bonferroni’s multiple comparisons tests).

When follow-up was conducted after the oral administration (*p.o.*) of abacavir, increased vascular permeability was observed in the auricle of B*57:01-Tg within 6 h (Fig. S6). This phenomenon was not seen when abacavir was administered to B*57:03-Tg or LM, suggesting that it was HLA-B*57:01-dependent. Treatment with 4-PB attenuated abacavir-induced vascular hyperpermeability in B*57:01-Tg (Fig. S6). Subsequent auricle tissue immunostaining performed 3 h after abacavir administration, revealed increased expression levels of phosphorylated IRE1α (p-IRE1α) and x-box binding protein 1 (XBP1) in the epidermis of B*57:01-Tg (Fig. 4a and b). Furthermore, 4-PB treatment suppressed the increase of p-IRE1α and XBP1 expression (Figs. 4a, b, and S7a, b). These results suggest that HLA-B*57:01-dependent ER stress responses in the epidermis can be triggered by systemic administration of abacavir. However, increased expression of p-IRE1α and XBP1 was not observed in the liver, kidney, or spleen (Fig. S7).

In a previous study, we reported that in B*57:01-Tg with PD-1 knockout (B*57:01-Tg/PD-1^−/−^) and CD4^+^ T cell depletion, continuous abacavir administration induced skin redness and infiltration of CD8^+^ T cells into the dermis of the auricular skin (47). These effects were suppressed by 4-PB treatment in three out of the five B*57:01-Tg/PD-1^−/−^ (Figs. 4c and S8). Elevation of serum CCL17 level, an indicator of skin inflammation (39), induced by continuous abacavir administration in CD4^+^ T cell-depleted B*57:01-Tg/PD-1^−/−^ was significantly suppressed by 4-PB treatment (Fig. 4d). These findings suggest that ER stress plays a role in HLA-B*57:01-dependent skin eruption caused by abacavir administration. However, the proportion of effector memory CD8^+^ T cells in the auricular lymph node did not change with or without 4-PB treatment following continuous abacavir *p.o.* administration in CD4^+^ T cell-depleted B*57:01-Tg/PD-1^−/−^ (Fig. 4e). This suggests that acquisition of adaptive immunity may occur independently of ER stress.

### Intracellular HLA aggregation and HLA binding with ER chaperones in HLA-B*57:01-expressing keratinocytes were induced by Abacavir

**Fig. 5.**
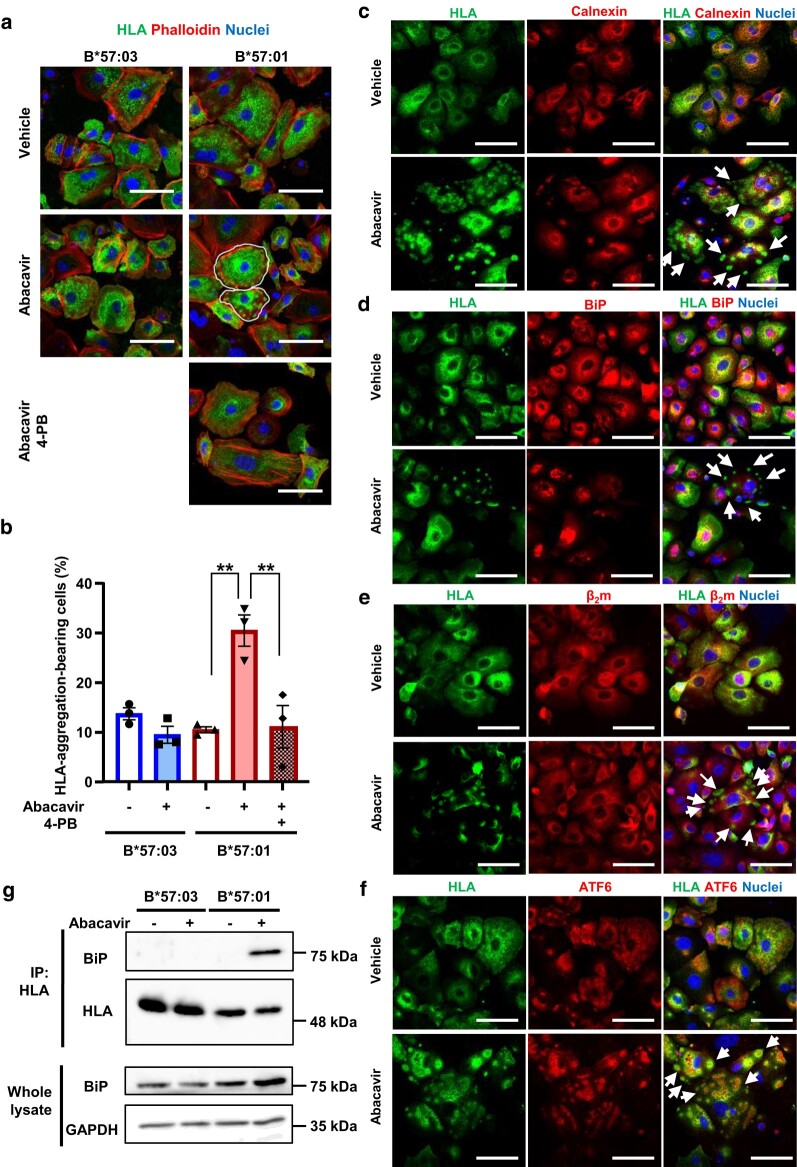
Abacavir induces intracellular HLA aggregation and misfolding in HLA-B*57:01-expressing keratinocytes. Panels a and b depict changes in FLAG-tagged HLA intracellular localization in keratinocytes from B*57:01-Tg (B*57:01-KCs) and B*57:03-Tg (B*57:03-KCs). After pretreatment with 4-PB, keratinocytes were incubated in the presence (+) or absence (−) of 100 μM abacavir for 30 min. Cells were stained with anti-FLAG antibody, rhodamine phalloidin for actin staining, and TO-PRO-3 for nucleus staining. Between 210 and 396 cells were observed for each experiment, and cells bearing FLAG-tagged protein (equivalent to HLA) aggregation are indicated by the white line. Each scale bar represents 50 μm. Error bars represent the mean ± SEM of three independent experiments. Significant differences (** *P* < 0.01) were observed compared to another group, as determined by one-way ANOVA followed by Bonferroni’s multiple comparisons tests. Panels c, d, e, and f show the localization of intracellular FLAG-tagged HLA aggregations in B*57:01-KCs, which were incubated in the presence or absence of 100 μM abacavir for 30 min. Cells were stained with anticalnexin (c), anti-BiP (d), anti-β_2_-microglobulin (β_2_m) (e), or anti-ATF6 (f), as well as anti-FLAG antibody and TO-PRO-3. Arrows indicate FLAG-tagged protein (equivalent to HLA) aggregations. Each scale bar represents 50 μm. Panel g presents the effects of abacavir on the formation of binding between HLA and BiP. Lysates from B*57:03-KCs and B*57:01-KCs, which were incubated in the presence (+) or absence (−) of 100 μM abacavir for 30 min, were subjected to immunoprecipitation (IP) of FLAG-tagged HLA. BiP and FLAG were detected by immunoblotting, with GAPDH used as the loading control.

In B*57:01-KCs, intracellular HLA aggregates were observed following abacavir exposure; in contrast, the formation of intracellular HLA aggregates was not increased in B*57:03-KCs or in HLA-B*57:01-transfected HeLa cells (Figs. 5a, b, and S9). The formation of HLA-B*57:01 intracellular aggregates was suppressed by 4-PB pretreatment (Fig. 5a and b). Intracellular HLA was mostly co-localized with calnexin and binding immunoglobulin protein (BiP), molecular chaperones in ER, suggesting that they were primarily localized in the ER (Fig. 5c and d). However, most intracellular HLA aggregates were not co-localized with calnexin or BiP (Fig. 5c and d). Most of the intracellular HLA co-localized with β_2_m, while HLA aggregates did not colocalize with β_2_m (Fig. 5e). This suggests that the HLA which formed aggregates did not bind to β_2_m. If misfolded proteins are present in the ER, BiP is released from ATF6. Subsequently, ATF6 relocates to the Golgi apparatus, where it undergoes cleavage, enabling its liberated N-terminal fragments to translocate into the nucleus (48–50). Here, we unexpectedly observed aggregates of ATF6 in abacavir-exposed B*57:01-KCs (Fig. S10) and their colocalization with aggregated HLA-B*57:01 (Fig. 5f). Immunoprecipitation of HLA revealed that BiP was bound to HLA at higher levels in abacavir-exposed B*57:01-KCs (Fig. 5g). When the expression level of HLA and intracellular localization of HLA in B*57:01-KCs with inhibition of de novo synthesis of HLA using cycloheximide were examined, abacavir exposure had no effect on the decrease of HLA in B*57:01-KC and the co-localization of HLA with calnexin (Fig. S11). These results suggest that HLA-B*57:01 becomes misfolded in the ER upon exposure to abacavir, leading to the formation of intracellular aggregates along with ATF6 outside the ER.

### HLA-B*57:01 bound to Abacavir during the maturation process within cells

**Fig. 6.**
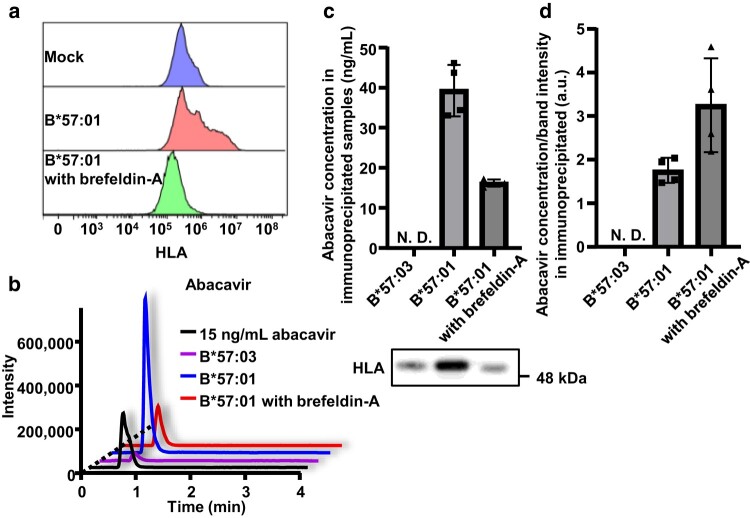
HLA-B*57:01 binds to abacavir during intracellular maturation. Panel a shows flow cytometric evaluation of HLA expression on the cell surface. HeLa cells, in which transfection of HLA-B*57:01 or B*57:03 and exposure to brefeldin-A were performed simultaneously, were subjected to flow cytometry for HLA assessment. Panels b, c, and d present LC-MS/MS chromatograms of standard and immunoprecipitated abacavir samples b), concentrations of abacavir and FLAG-tagged HLA expression in FLAG-immunoprecipitated samples c), and abacavir concentration per unit of immunoprecipitated FLAG-tagged HLA in FLAG-immunoprecipitated samples d). Lysates (containing 1.5 mg of protein) from HeLa cells, in which transfection of HLA-B*57:01 or B*57:03 and exposure to brefeldin-A as well as 100 μM of abacavir were performed simultaneously, were subjected to immunoprecipitation of FLAG-tagged HLA. A total of 120 μL of immunoprecipitated samples were obtained. FLAG-tagged HLA expression in FLAG-immunoprecipitated samples was assessed via immunoblotting and analyzers. N.D. indicates “not detected” c) and “not determined” d).

Since we observed an HLA polymorphism-dependent intracellular response to abacavir, we hypothesized that HLA could bind to abacavir intracellularly. Although not yet confirmed, the abacavir parent drug has been predicted to interact with HLA-B*57:01 in the ER (21, 22). Consistent with a previous study (20), we demonstrated the binding of HLA-B*57:01 to abacavir using HeLa cells in which HLA was transiently expressed (36). To clarify the intracellular binding of abacavir to HLA-B*57:01, we restricted HLA expression on the cell surface by simultaneously exposing the cells to brefeldin-A along with HLA introduction (Fig. 6a). Abacavir binding to HLA-B*57:01 was detected in HeLa cells treated with brefeldin-A (Fig. 6b and c), and the amount of abacavir associated with HLA was not less than that in the absence of brefeldin-A (Fig. 6d). This finding implies that the rapid intracellular responses observed in HLA-B*57:01-expressing keratinocytes following abacavir exposure is attributed to the intracellular binding of the HLA molecule to abacavir.

## Discussion

The development of HLA polymorphism-dependent SCARs is hypothesized to primarily result from the effect of drugs on antigen presentation to T cells on the cell surface (1) (6). Therefore, initial expectations did not consider the possibility of an HLA polymorphism-dependent drug response occurring in an experiment devoid of T cells. However, this study presents an intriguing finding: a specific immunological response to abacavir occurs solely in keratinocytes and is influenced by HLA polymorphism.

**Fig. 7.**
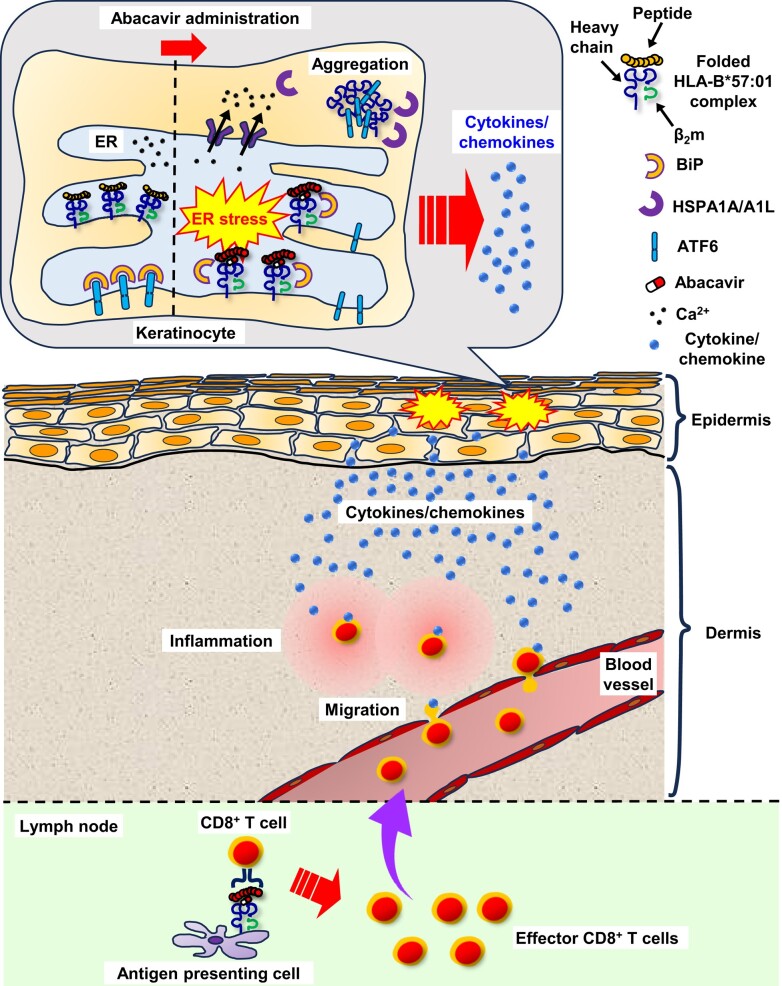
Proposed mechanism underlying abacavir-induced HLA polymorphism-specific responses in keratinocytes during the early stage of idiosyncratic drug-induced skin eruption. In addition to abacavir’s effect on antigen presentation by HLA, abacavir’s effect on the intracellular behavior of HLA can be responsible for the development of abacavir-induced skin eruption. The formation of the tetrameric complex of HLA-B*57:01-abacavir-β_2_-microglobulin (β_2_m)-peptide within the ER needs further consideration.

Subsequently, we explored the mechanisms underlying these responses. We observed an immediate reaction in keratinocytes, characterized by increased calcium release in response to abacavir, and this response was dependent on HLA polymorphism (Fig. 3a). Furthermore, expression levels of CHOP were increased in abacavir-exposed keratinocyte in HLA polymorphism-dependent manner (Fig. 3b). These findings suggest the occurrence of HLA polymorphism-dependent ER stress. Furthermore, we found that keratinocytes, in an HLA polymorphism-dependent manner, increased cytokine production and T lymphoblast migration (Fig. 3c–i). These effects were counteracted by 4-PB, suggesting that the immunological activation of keratinocytes in response to ER stress led to the recruitment of inflammatory cells into the skin (Fig. 3c–i). Interestingly, while the activation of CD8^+^ T cells in the lymph nodes remained unaffected by the suppression of ER stress following oral administration of abacavir to B*57:01-Tg/PD-1^−/−^, skin eruption and elevated serum CCL17 levels were suppressed (Fig. 4). This implies that the activation of CD8^+^ T cells still occurs based on the effect of abacavir on antigen presentation, consistent with previous studies (21, 22). However, abacavir-induced recruitment of inflammatory cells to the skin was reliant on HLA polymorphism-dependent ER stress responses in keratinocytes. ER stress is typically triggered by protein misfolding and aggregation. Exposure of keratinocytes to abacavir resulted in increased intracellular aggregation of HLA-B*57:01 without β_2_m and binding of BiP to HLA-B*57:01 (Fig. 5), indicating misfolding of HLA in the ER, which likely contributed to ER stress (48, 49). Additionally, abacavir affects HLA during the maturation process within cells (Fig. 6). A series of observations indicate that the presence of abacavir in the ER can induce intracellular aggregation and misfolding of HLA-B*57:01 in keratinocytes, leading to an ER stress response that immunologically activates the keratinocytes and triggers T cell infiltration into the skin tissue, resulting in skin eruption (Fig. 7).

This study revealed an immediate response mediated by drug-induced HLA polymorphism-dependent ER stress. Additionally, HLA polymorphism-dependent drug hypersensitivity has been attributed to acquired immunity (1), which is amplified by various factors, including the activation of innate immunity and stress responses (31, 33, 51). Therefore, the ER stress responses observed in keratinocytes in our study may be part of the pathway leading to acquired immunity. Abacavir exposure has been shown to immediately induce HSP70-mediated innate immune responses in PBMCs of patients with AHS (25). In PBMCs of patients with AHS exposed to abacavir, an increase in intracellular expression of HSP70 was observed, along with the presence of HSP70-positive vesicles or aggregates (25), consistent with our results. Although the upstream mechanism initiating this series of events was not clarified, ER stress in specific PBMC cell types could play a role upstream of HSP70 elevation and redistribution, given ER stress in HLA-B*57:01-expressing keratinocytes increased HSP70 mRNA expression in this study (Fig. 2).

Various genetic backgrounds, both directly and indirectly involved in the maturation process of HLA, are associated with hypersensitivity reactions. Mutations in HSP70 and proteasome subunits are linked to the development of drug-induced SJS/TEN (52, 53), and mutations in the molecular chaperone HSP70-Hom are associated with AHS (24). In drug-triggered eruptions involving HLA polymorphisms, ERAP1 mutations are correlated with AHS in HLA-B*57:01 carriers, and ERAP2 mutations have been associated with nevirapine-induced SJS/TEN in HLA-C*04:01 carriers (26, 54). Since ERAP1 and ERAP2 are genes involved in HLA complex stability (55–57), their mutations may induce drug-induced HLA misfolding and facilitate ER stress. We previously demonstrated that HLA molecules associated with ADRs exhibit a low affinity for β_2_m and tend to accumulate in the ER, suggesting a propensity for HLA misfolding in the ER or an ER stress response triggered by drugs (58). As shown in Fig. 6, drugs may affect HLA during the maturation process within cells. A genetic background predisposed to ER stress and decreased stability of the HLA complex could collectively contribute to the development of drug eruptions.

Examining factors that influence the stability of the HLA complex can provide insights into both interindividual variability in HLA-mediated drug-induced ER stress responses and toxicity, as well as mechanisms underlying tissue-specific differences. We observed an ER stress response in keratinocytes and the epidermis, while other cell types, such as hepatocytes and HeLa cells, as well as certain tissues, did not exhibit a comparable response to abacavir (Figs. 3, 4, S3, and S7). Although abacavir binds to HLA-B*57:01 in various cell types (20–22), its exposure in most cell types was expected to induce HLA-B*57:01 misfolding and a subsequent ER stress response; however, our findings contradict this expectation. To reconcile this contradiction, we propose that while the HLA-B*57:01 complex generally shows increased instability and misfolding in the ER upon exposure to abacavir, keratinocytes may have an inherent predisposition toward HLA structural instability and misfolding, ultimately leading to HLA-B*57:01 misfolding and ER stress response.

The assembly and structural stability of the HLA complex in the ER depend on proteins constituting the peptide-loading complex (PLC), such as transporters associated with antigen presentation (TAP) 1, tapasin, and TAP binding protein-like (TAPBPL) (59). The expression levels of these PLC proteins vary across different tissues and cell types. For instance, proteins like tapasin, TAPBPL, and ERAP1 have lower expression levels in keratinocytes compared to hepatocytes, indicating a potential difference in HLA complex assembly (Human Protein Atlas, https://www.proteinatlas.org/). Indeed, we observed an HLA polymorphism-dependent intracellular immunological response to abacavir solely in keratinocytes (Fig. 1) and not in hepatocytes or other cell types (Fig. S3). Additionally, the ER stress response observed in the epidermis was not replicated in the liver (Fig. S7). These findings suggest that variations in PLC protein expression may underlie the tissue specificity of HLA-mediated drug-induced ER stress responses. This implies that differences in PLC composition could explain why ADRs involving HLA class I are more likely to manifest as conditions like SCARs rather than liver, kidney, or blood disorders (1, 2, 14, 15).

This study revealed the aggregation and binding of abacavir to HLA-B*57:01 and its interaction with BiP (Fig. 5). When misfolded proteins accumulate in the ER, BiP dissociates from ER stress sensors such as IRE1 and ATF6, acting as a chaperone for the misfolded proteins. This triggers an ER stress response (48, 49, 50, 60). The observed binding of BiP to HLA in this study suggests that HLA misfolding occurs in the ER, leading to an ER stress response. In this study, HLA aggregates exhibited minimal colocalization with calnexin or BiP but did colocalize with aggregated ATF6 (Fig. 5). This suggests that, in the presence of abacavir, HLA migrates from the ER along with ATF6 as vesicles bud from the ER, subsequently forming intracellular aggregates. If ATF6 aggregates intracellularly after budding from the ER, homeostasis through ATF6 signaling may be disrupted, resulting in enhanced ER stress (61, 62). However, the exact mechanisms underlying the generation of these aggregates remain unclear and warrant further investigation.

This study underscores the association between abacavir and HLA-B*57:01-mediated AHS, specifically implicating HLA polymorphism-dependent ER stress. Intracellular binding of abacavir to HLA-B*57:01 was demonstrated, serving as the potential trigger for the HLA-dependent abacavir-induced intracellular response. Our discovery that HLA has the potential to induce ER stress through binding with drugs within the cell can provide significant pharmacological and physiological insights, complementing its well-known role in promoting immune activation through binding with TCR on the cell surface.

## Materials and methods

### Materials

Abacavir was purchased from Carbosynth, Ltd. (Compton, Berkshire, UK). 4-PB, 4μ8c, ceapin-A7, GSK2606414, anti-FLAG M2 magnetic beads, and anti-FLAG mAb (M2) were acquired from Sigma-Aldrich (St. Louis, MO, USA). Anti-CD4 monoclonal antibody (mAb; GK1.5), PE-Cy7 anti-mCD8a mAb (53–6.7), FITC anti-mCD62L mAb (MEL-14), PE antimouse/human CD44 mAb (IM7), and FITC antihuman HLA-A, B, C mAb (W6/32) were sourced from BioLegend (San Diego, CA, USA). Sodium 4-PB, acyclovir, and brefeldin-A were obtained from Tokyo Chemical Industry (Tokyo, Japan). Accutase, Dulbecco’s Modified Eagle Medium (DMEM), MEM, and antibiotic–antimycotic mixed solution were purchased from Nacalai Tesque (Kyoto, Japan). Anti-ATF6 polyclonal antibody (pAb) (ab37149), anticalnexin pAb (ab22595), anti-XBP1 pAb (ab37152), anti-BiP pAb (ab21685), anti-CD8 mAb (YTS169.4), and antimouse or rat IgG conjugated with Alexa Fluor 488 were procured from Abcam (Cambridge, UK). Anti-β_2_m polyclonal anitibody was purchased from GENETEX (Irvine, CA, USA). Anti-GAPDH mAb (D16H11) was bought from Cell Signaling Technology (Beverly, MA, USA). Anti-Phospho-IRE1 alpha (Ser724) pAb (PA5-105424), antirabbit IgG conjugated with FITC or Alexa Fluor 546, TO-PRO-3, rhodamine phalloidin, Hoechst 33342, and fetal bovine serum were obtained from Thermo Fisher Scientific (Waltham, MA, USA). HaCaT cells were purchased from Cell Lines Service (Eppelheim, Germany). HeLa and EL4 cells were acquired from RIKEN Cell Bank (Tsukuba, Japan).

### Animals

HLA-Tg, their LMs, B*57:01-Tg/PD-1^−/−^, and PD-1^−/−^ were generated as described previously (36, 37). We used 1- or 2-day-old mice to prepare primary mouse KCs. In 8- to 16-week-old mice, abacavir was orally administered daily at doses of 20 or 30 mg/body, suspended in a 1% carboxymethyl cellulose aqueous solution, for 1 or 5 days. Anti-CD4 mAb (0.25 mg/body) was administered intraperitoneally on days 3 and 1 for CD4^+^ T cell depletion (37). We performed 4-PB pretreatment using daily intraperitoneal injections (1 day before day 0 and 30 min before each abacavir administration) of 4 mg/body of sodium 4-PB. Animals were treated humanely in accordance with guidelines issued by the National Institutes of Health. All procedures were approved by the Animal Care Committee of Chiba University. Serum CCL17 levels were measured using a Mouse CCL17/TARC DuoSet enzyme-linked immunosorbent assay kit (R&D Systems, Inc., Minneapolis, MN, USA). Effector memory CD8^+^ T cells (CD44^high^ CD62L^low^) in auricular lymph nodes were measured using flow cytometry with an EC800 flow cytometer (Sony; Tokyo, Japan) and analyzed using Flow Logic software (Bay Bioscience Co., Ltd., Tokyo, Japan), as described previously (36, 37).

### Preparation of mouse primary keratinocytes

The keratinocytes used in this study were prepared as previously described (63), with minor modifications. Skin tissue was isolated from 1- or 2-day-old mice and incubated for 15–18 h at 4 ° C in CnT-PR medium (CELLnTEC; Bern, Switzerland) supplemented with antibiotic–antimycotic mixed solution and 5 mg/mL dispase (Wako Pure Chemical, Osaka, Japan). Subsequently, the epidermis was separated from the dermis, and keratinocytes were dissociated from the epidermis using Accutase. Postcollection, the keratinocytes were suspended in CnT-PR medium with antibiotic–antimycotic mixed solution and plated at a density of 3 × 10^5^ cells/well in 12-well plates (Greiner Bio-One, GmbH, Germany) coated with collagen. The cells were cultured at 37 ° C in a 5% CO_2_ atmosphere. The culture medium was changed 16–20 h after plating (on day 3), and the cells were assayed on day 6.

### DNA microarray analysis

Total RNA was isolated using Sepasol-RNA I Super G (Nacalai Tesque), and 2 µg of the sample was subjected to DNA microarray analysis using the “3D-Gene” mouse oligo chip 24k (Toray Industries Inc., Tokyo, Japan). Gene expression levels were quantified based on the intensity of Cy3 signals. The cutoff value for global normalization was set at ≤ 1.2. Enrichment analyses were conducted using GO analysis and KEGG pathways analysis and carried out with gene set enrichment analysis software ver. 4.0.1, focusing on 1.5-fold changes in gene expression in B*57:01-KCs compared to B*57:03-KCs.

### HaCaT and HeLa cultures and introduction of HLA

HaCaT cells were maintained in CnT-PR media supplemented with an antibiotic–antimycotic solution. HeLa cells were maintained as previously described (58). The cells were cultured at 37 ° C in a 5% CO_2_ atmosphere. mRNA produced by in vitro transcription of pcDNA vectors expressing HLA or lacZ, using the mMESSAGE mMACHINE T7 Ultra kit (Thermo Fisher Scientific, Waltham, MA, USA), was transfected using Lipofectamine MessengerMAX (Thermo Fisher Scientific). Simultaneously, the cells were exposed to abacavir.

### Calcium release imaging

Keratinocytes were preincubated with 10 µM Cal-520 AM (AAT Bioquest; Sunnyvale, CA, USA) and 0.02% Pluronic F127 for 2 h. Subsequently, the medium was changed, and the keratinocytes were exposed to 100 µM abacavir. All samples were imaged using either a BZ-X700 microscope (Keyence, Osaka, Japan) or a Zeiss LSM 780 confocal microscope (Carl Zeiss, Jena, Germany). Images were analyzed using ImageJ software from the National Institutes of Health.

### Immunocytochemistry

Cells were fixed in 4% paraformaldehyde, permeabilized with 0.05% Triton X-100, and stained with anti-ATF6 pAb, anti-β_2_m pAb, anti-FLAG mAb, anti-BiP pAb, and anticalnexin pAb followed by incubation with antibodies against mouse IgG conjugated with Alexa Fluor 488, as well as rabbit IgG conjugated with Alexa Fluor 546 or FITC, rhodamine phalloidin, and TO-PRO-3. All samples were imaged using a Zeiss LSM 700 confocal microscope equipped with Airyscan and ZEN software (Carl Zeiss). A minimum of 180 cells were scored in each experiment to determine the relative expression of ATF6/HLA-aggregation-bearing cells. Blind tests were performed by randomizing the samples prior to staining.

### Detection of mRNA level changes

One hundred micromolar of abacavir was dissolved in cultured medium and exposed for 0, 3, 6, 12 h for measurement of mRNA levels of CHOP. Abacavir was dissolved in CnT-PR-D (CELLnTEC) and exposed for 12 h after removing the culture medium for measurement of mRNA levels of cytokines, chemokines, Hspa1a, Hspa1 l, Tmem129, Ube2j1, Itpr1, Pdgfra, and K16. Pretreatment with 4-PB (5 mM) and UPR inhibitors (4μ8c 10 μM; ceapin-A7 10 μM; GSK2606414 0.5 μM) were conducted by directly dissolving the compound in the culture medium 3 h before abacavir exposure. Total RNA was isolated using Sepasol-RNA I reagent (Nacalai Tesque) and reverse-transcribed using ReverTra Ace qPCR RT Master Mix (TOYOBO; Osaka, Japan). The resulting cDNA was mixed with THUNDERBIRD SYBR qPCR Mix (TOYOBO) and subjected to real-time PCR using a LightCycler Nano System (Roche, Mannheim, Germany). Relative mRNA expression levels were calculated after normalization against GAPDH mRNA levels. The oligonucleotide sequences for mRNA target are shown in Table S2.

### Histology and immunohistochemistry

After administering abacavir or vehicle, the auricle, liver, kidney, and spleen were embedded in Tissue-Tek O.C.T. compound and sectioned. We conducted hematoxylin and eosin (H&E) staining and CD8 histology and immunohistochemistry using a previously reported method (37). For staining the ER stress markers p-IRE1α and XBP1, the sliced tissues were fixed in 4% paraformaldehyde, permeabilized with 0.2% Triton X-100, and stained with rabbit anti-p-IRE1α or XBP1 antibodies, followed by incubation with an antibody against rabbit IgG conjugated with Alexa Fluor 546 and Hoechst 33342. All samples were imaged using a BZ-X700 microscope.

### Immunoprecipitation and immunoblotting

Keratinocytes or HeLa cells were lysed in NP-40 buffer (0.5% NP-40, 150 mM NaCl, 50 mM Tris-HCl, pH 8.0). Subsequently, the whole cell lysate was immunoprecipitated using anti-FLAG M2 magnetic beads; bound proteins were then eluted with 0.1 M glycine-HCl (pH 3.0). Both immunoprecipitates and whole cell lysate proteins were separated using SDS-PAGE and transferred to polyvinylidene fluoride membranes (Merck Millipore, Berlin, Germany). After blocking the membranes, blots were incubated with primary antibodies. Immunolabeled proteins were detected using a horseradish peroxidase-labeled secondary antibody (Cytiva, Tokyo, Japan) and ECL Prime detection reagent (GE Healthcare, Buckinghamshire, UK), utilizing a LAS-4000 luminescent image analyzer (GE Healthcare). The density of the detected bands was measured using ImageJ software.

### Chemotaxis assay with EL4 cells and keratinocytes

EL4 cells were maintained in Dulbecco’s Modified Eagle Medium (DMEM) supplemented with 10% fetal bovine serum and an antibiotic–antimycotic mixed solution. Chemotaxis assays were performed using a coculture of EL4 cells (stained with DiI from Wako Pure Chemical and suspended in CnT-PR-D; 5 × 10^5^ cells/well) in cell culture inserts with 8.0 μm pores (BM Equipment Co. Ltd., Tokyo, Japan) and keratinocytes (in medium changed to CnT-PR, with or without abacavir) in 12-well plates. Forty-eight hours later, chemotaxis activity was assessed using DiI fluorescence of the culture medium in the lower chambers. Cells were cultured at 37 ° C in a humidified atmosphere containing 5% CO_2_ in air.

### Exposure of abacavir to HeLa cells in which HLA-B*57:01 remains internalized

HeLa cells were plated in flat-bottomed 12-well plates at a density of 1.0 × 10^5^ cells/well. After 24 h, the cells were transfected with a FLAG-tagged HLA-B*57:01 expression pcDNA vector using Lipofectamine 2000 (Thermo Fisher Scientific). Simultaneously, the cells were exposed to 250 ng/mL of brefeldin-A and 100 μM abacavir. After 12 h, the cells were dissociated using Accutase. Expression levels of HLA on the cell surface were then measured using flow cytometry with an EC800 flow cytometer and analyzed using FlowLogic software, as described previously (58). Alternatively, the cells were lysed in NP-40 buffer and immunoprecipitated.

### Determination of abacavir concentrations

To prepare samples for liquid chromatography-tandem mass spectrometry (LC-MS/MS), 100 μL of each sample was added to 10 ng of acyclovir as an internal standard. This mixture was then combined with 1 mL of methanol and centrifuged at 12,000 × *g* for 10 min. The supernatants were evaporated, and the residues were reconstituted in 1 mL of ultrapure water. These were then applied to an Oasis HLB cartridge (Waters, Milford, MA, USA) for sample preparation and solid-phase extraction. Subsequently, the samples were evaporated again, and the residues were dissolved in 100 μL of the LC mobile phase. Aliquots (10 μL) were injected into the LC-MS/MS system, which consisted of an LC system (Prominence, Shimadzu Corporation, Kyoto, Japan) and a tandem mass spectrometer (QTRAP 4500, Sciex, Framingham, MA, USA). Chromatography was performed using a YMC-Triart C18 column (50 mm × 2.0 mm internal diameter, 3 μm particle size, YMC Co., Ltd, Kyoto, Japan). A column oven was set at 40 °C. The mobile phase consisted of water with 0.1% formic acid (A) and acetonitrile with 0.1% formic acid (B). A constant elution rate of 0.2 mL/min was maintained in gradient mode as follows: 0–0.2 min (50% mobile phase B), 0.2–6.0 min (linear increase to 95% B), 6.0–8.5 min (95% mobile phase B), and 8.6–11.0 min (re-equilibration to 50% B). The MS scan operated in positive ionization mode. The detection molecular ions selected for abacavir were precursor 287.166 m/z and product 199 m/z with a 27 V collision energy, while for acyclovir they were precursor 226.1 m/z and product 184 m/z with a 17 V collision energy. The areas obtained for abacavir were normalized to those obtained for acyclovir.

### Statistical analysis

All data are presented as mean ± SD or SEM GraphPad Prism 9 software (GraphPad Software, La Jolla, CA, USA) was used for all statistical analyses. Data were analyzed using Student’s t test, and for multiple comparisons, either Bonferroni’s test or Dunnett’s test was applied following one-way ANOVA. In all instances, a *P*-value of < 0.05 was considered statistically significant. All experiments have been replicated at least 3 times and are reproducible.

## Supplementary Material

pgae140_Supplementary_Data

## Data Availability

All data are included in the manuscript and Supplementary material.
