# Supplementary material for: HLA-B*57:01-dependent intracellular stress in keratinocytes triggers dermal hypersensitivity reactions to abacavir
Source: PNAS Nexus. 2024 Apr 2;3(4):pgae140. doi: 10.1093/pnasnexus/pgae140 (PMC11018537; doi:10.1093/pnasnexus/pgae140)
Supplement: pgae140_Supplementary_Data [file pgae140_supplementary_data.zip › PNASNEXUS-PNASNEXUS-2024-00333-T-s01.pdf]

PNAS nexus

Supplementary Information for
HLA-B*57:01-dependent intracellular stress in keratinocytes triggers dermal hypersensitivity reactions to abacavir

Akira Kazaoka, Sota Fujimori, Yushiro Yamada, Tomohiro Shirayanagi, Yuying Gao, Saki Kuwahara, Naoki Sakamoto, Takeshi Susukida, Shigeki Aoki*, Kousei Ito*

Laboratory of Biopharmaceutics, Graduate School of Pharmaceutical Sciences, Chiba University, 1-8-1 Inohana, Chuo-ku, Chiba-city, Chiba 260-8675, Japan.

*Corresponding authors:
Shigeki Aoki, Ph.D.
Laboratory of Biopharmaceutics, Graduate School of Pharmaceutical Sciences, Chiba University, 1-8-1 Inohana, Chuo-ku, Chiba-city, Chiba 260-8675, Japan.
Tel: +81 43 226 2888, Fax: +81 43 226 2888, E-mail: aokishigeki@chiba-u.jp

Kousei Ito, Ph.D.
Laboratory of Biopharmaceutics, Graduate School of Pharmaceutical Sciences, Chiba University, 1-8-1 Inohana, Chuo-ku, Chiba-city, Chiba 260-8675, Japan.
Tel: +81 43 226 2886, Fax: +81 43 226 2886, E-mail: itokousei@chiba-u.jp

This PDF file includes:

Supporting text
Figures S1 to S11
Table S1 and S2
Legends for Movie S1
SI References

Other supporting materials for this manuscript include the following:

Movies S1

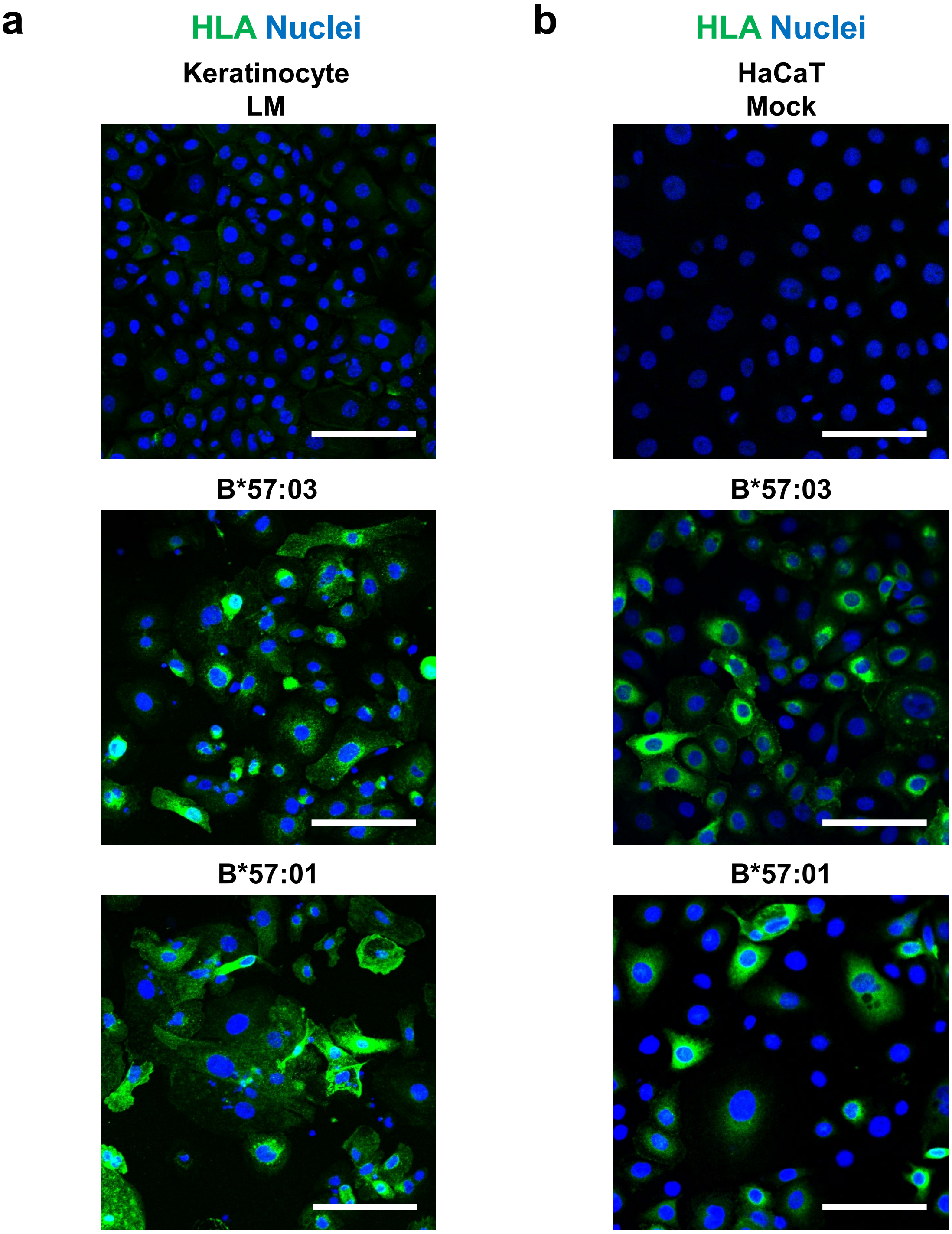

Fig. S1. Expression of HLA in keratinocytes of HLA-Tg and HLA transfected HaCaT cells. **a** Expression of introduced FLAG-tagged HLA in keratinocytes of B*57:01-Tg, their littermates, and B*57:03-Tg. **b** Expression of FLAG-tagged HLA in HLA-B*57:01-, B*57:03-, or mock mRNA-transfected HaCaT cells. Keratinocytes and HaCaT cells were stained with anti-FLAG antibody (green) and TO-PRO®-3 (blue; nucleus staining). Each scale bar represents 100 µm.

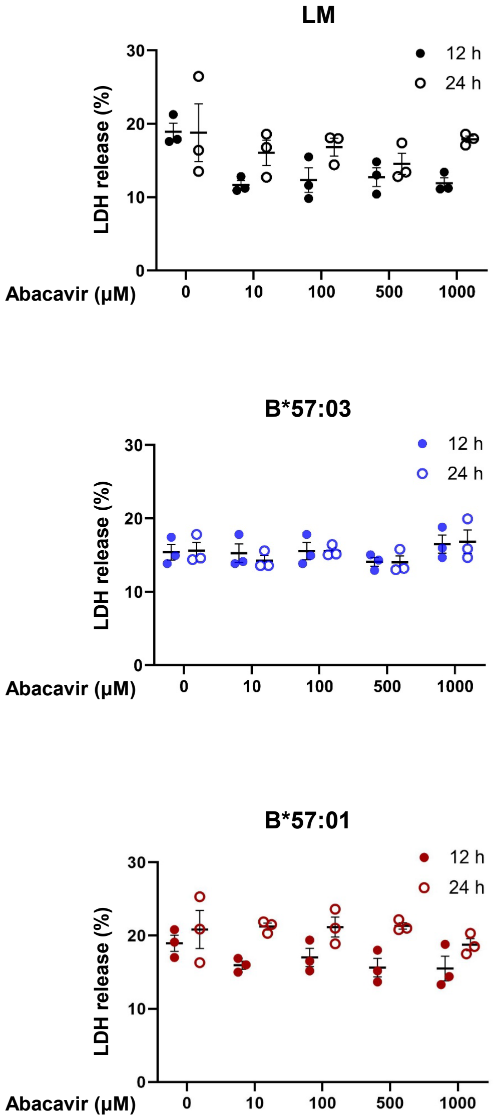

Fig. S2 Lactate dehydrogenase (LDH) leakage from abacavir-exposed keratinocytes. Cytotoxicity was measured using LDH release at 12 h and 24 h after the exposure to abacavir in B*57:01-KCs, LM-KCs, and B*57:03-KCs. The degree of LDH release was expressed as a percentage of maximum LDH release in the medium from control keratinocytes treated with Triton X-100 for 24 h. Data are expressed as the mean ± S.E.M. (n = 3/group).

Measurement of LDH

Cytotoxicity was assessed by measuring the release of LDH using the TaKaRa LDH cytotoxicity detection kit (TaKaRa Bio Inc., Kusatsu, Japan), according to the manufacturer's instructions. Absorbance was measured using a plate reader (Multiskan JX; Labsystems, Vienna, VA) at 492 nm. LDH release was calculated as follows: (% control) = (experiment – "low control")/("high control" – "low control") × 100%. "Low control" represents LDH released from cells treated with Milli Q water and "high control" represents LDH released from cells treated with 1% Triton X-100.

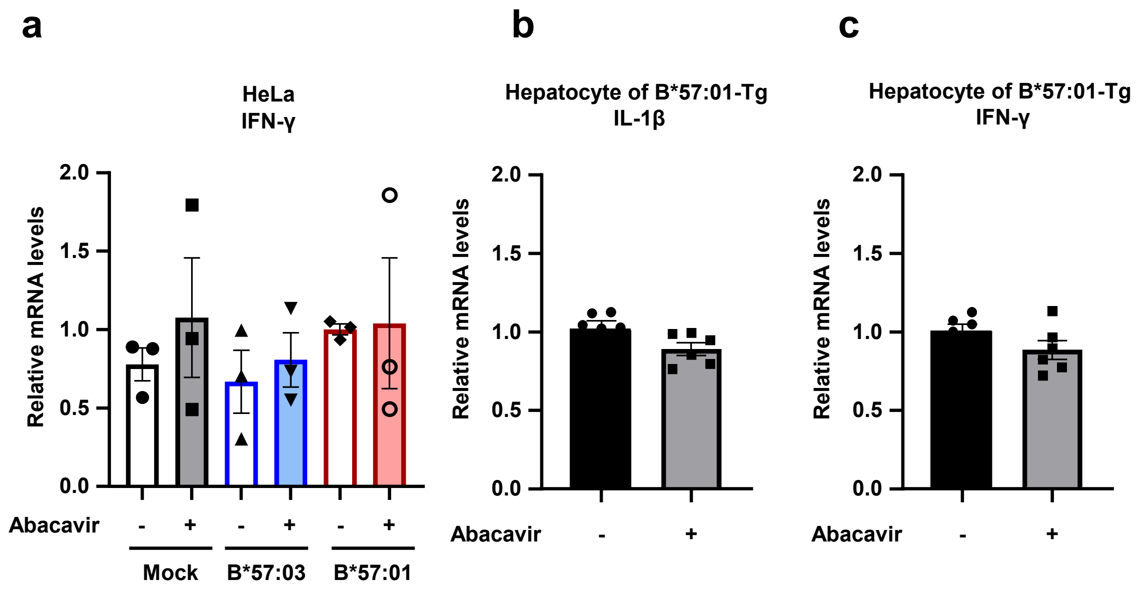

Fig. S3. Effects of abacavir on mRNA expression levels of cytokines in HLA-transfected HeLa cells and hepatocytes from HLA-B*57:01-Tg. **a** mRNA expression levels of IFN-γ relative to GAPDH in HLA-B*57:01-, B*57:03-, or mock-transfected HeLa cells. HLA-transfected HeLa cells was incubated in the presence or absence of 100 μM abacavir for 12 h. Data are expressed as the mean ± S.E.M. (n = 3/group). There were no significant differences (t-tests). **b, c** mRNA expression levels of IL-1β (**b**) and IFN-γ (**c**) relative to GAPDH in hepatocytes. Hepatocytes were incubated in the presence of or absence of 100 μM abacavir for 12 h. Data are expressed as the mean ± S.E.M. (n = 6/group). There were no significant differences (t-tests).

Preparation of mouse primary hepatocytes

Mouse hepatocytes were isolated using a two-step perfusion method as reported previously[1]. Hepatocytes were suspended in a plating medium consisting of Williams' Medium E (Thermo Fisher Scientific; Waltham, MA, USA) supplemented with antibiotic-antimycotic, 5% fetal bovine serum, 4 μg/mL insulin, 1 μM dexamethasone, GlutaMAX (Thermo Fisher Scientific), and 15 mM HEPES (pH 7.4). Hepatocyte suspensions were plated onto 12 plates precoated with collagen type I at a density of 3.5×10^5 cells/well. After an initial attachment period of 2 h, the medium was replaced with culturing medium (Williams' Medium E supplemented with antibiotic-antimycotic, ITS premix, 1 μM dexamethasone, and GlutaMAX) with/without abacavir for 12 h. The cells were cultured at 37 °C in a 5% CO2 atmosphere.

a

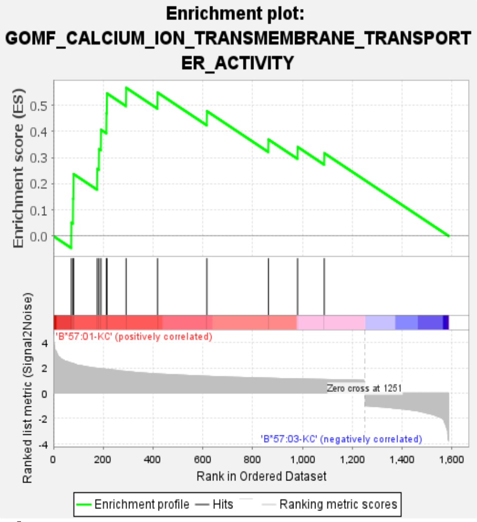

GOMF Calcium Ion Transmembrane Transporter Activity	
Gene Symbol	Fold Change
Ryr2	2.82
Pkdrej	2.77
Itpr1	2.75
Trpv6	2.29
Atp2c2	2.28
Htr1b	2.26
Tmc2	2.21
Cacng2	2.20
Oprm1	2.04
Scn3a	1.86
Cacng4	1.72
Cacna1d	1.61
Atp2b4	1.57
Trpc4	1.54

b

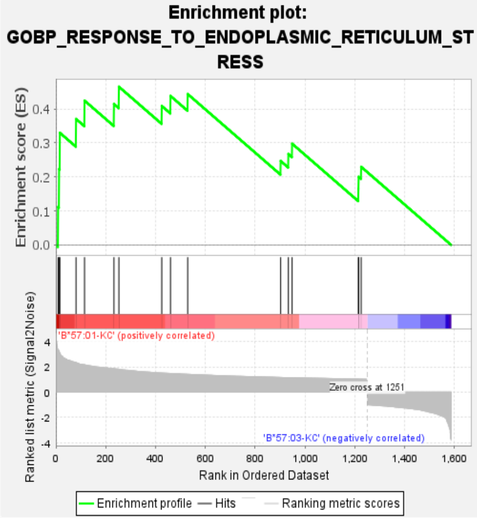

GOBP Response to Endoplasmic Reticulum Stress	
Gene Symbol	Fold Change
Hspa1a	5.12
Tmem129	4.69
Ube2j1	4.25
Itpr1	2.75
Tardbp	2.17
Calr3	2.12
Nod2	1.86
Sirt1	1.83
Thbs1	1.78
Ern1	1.60
Clu	1.59
Tmem67	1.58
Grina	1.51
Crebzf	1.51
Bak1	1.51

c

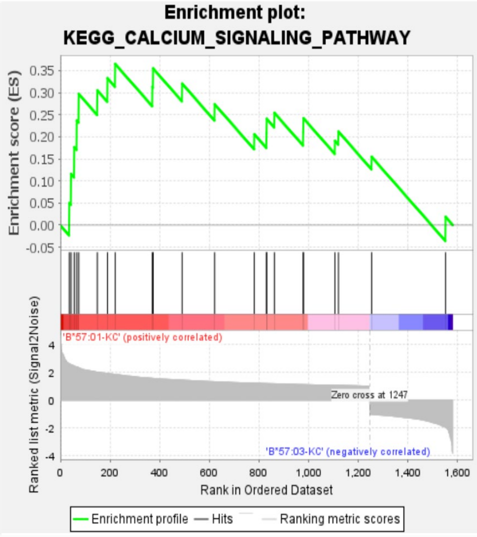

Calcium Signaling Pathway	
Gene Symbol	Fold Change
P2rx6	3.20
Pdgfra	3.04
Pde1a	2.93
Ryr2	2.82
Itpr1	2.75
Adcy1	2.34
Hrh2	2.24
Erbb2	2.18
Htr2b	1.91
Tacr2	1.90
Ednrb	1.81
Adcy4	1.72
Ptgfr	1.62
Adcy9	1.62
Cacna1d	1.61
Atp2b4	1.57
Plcg2	1.57
Tacr3	1.54
Adrb2	1.54

d

Chemokine Signaling Pathway	
Gene Symbol	Fold Change
Ccl7	2.65
Wasl	2.54
Adcy1	2.34
Arrb1	1.95
Ccl27a	1.73
Adcy4	1.72
Ccl3	1.66
Adcy9	1.62
Cxcl12	1.61
Gngt1	1.59
Ccr9	1.59
Cxcl10	1.56
Crkl	1.55
Cx3cr1	1.51

e

Cytokine-Cytokine Receptor Interaction	
Gene Symbol	Fold Change
Il18rap	13.37
Pdgfra	3.04
Ccl7	2.65
Tnfsf4	2.20
Il2	1.95
Kit	1.94
Ngfr	1.84
Tnfrsf11b	1.76
Ccl27a	1.73
Ifna2	1.72
Tnf	1.68
Csf3r	1.66
Ccl3	1.66
Il21	1.65
Prlr	1.62
Cxcl12	1.61
Il9r	1.61
Ccr9	1.59
Il2ra	1.59
Tnfrsf17	1.56
Cxcl10	1.56
Il1rap	1.54
Flt3	1.54
Cx3cr1	1.51
Il17b	1.50

Fig. S4. Comprehensive mRNA expression analysis uncovers HLA polymorphism-dependent responses to abacavir in keratinocytes. (**a–c**) Gene-set enrichment analysis presents representative enrichment plots for “KEGG Calcium Signaling Pathway”, “GOMF Calcium Ion Transmembrane Transporter Activity”, and “GOBP Response to Endoplasmic Reticulum Stress”, and gene lists with ≥ 1.5-fold higher expression are registered in these gene sets in abacavir-exposed keratinocytes of

B*57:01-Tg compared to B*57:03-Tg. The x-axis represents the ranked gene list in descending order based on log_2-transformed mRNA expression levels relative to B*57:03-KCs, and the y-axis represents the running sum of the enrichment score (top) and log_2-transformed mRNA expression levels for each gene (bottom). (**d, e**) Gene lists with ≥ 1.5-fold higher expression are registered in “Chemokine Signaling Pathway” and “Cytokine-Cytokine Receptor Interaction” in abacavir-exposed keratinocytes of B*57:01-Tg compared to B*57:03-Tg.

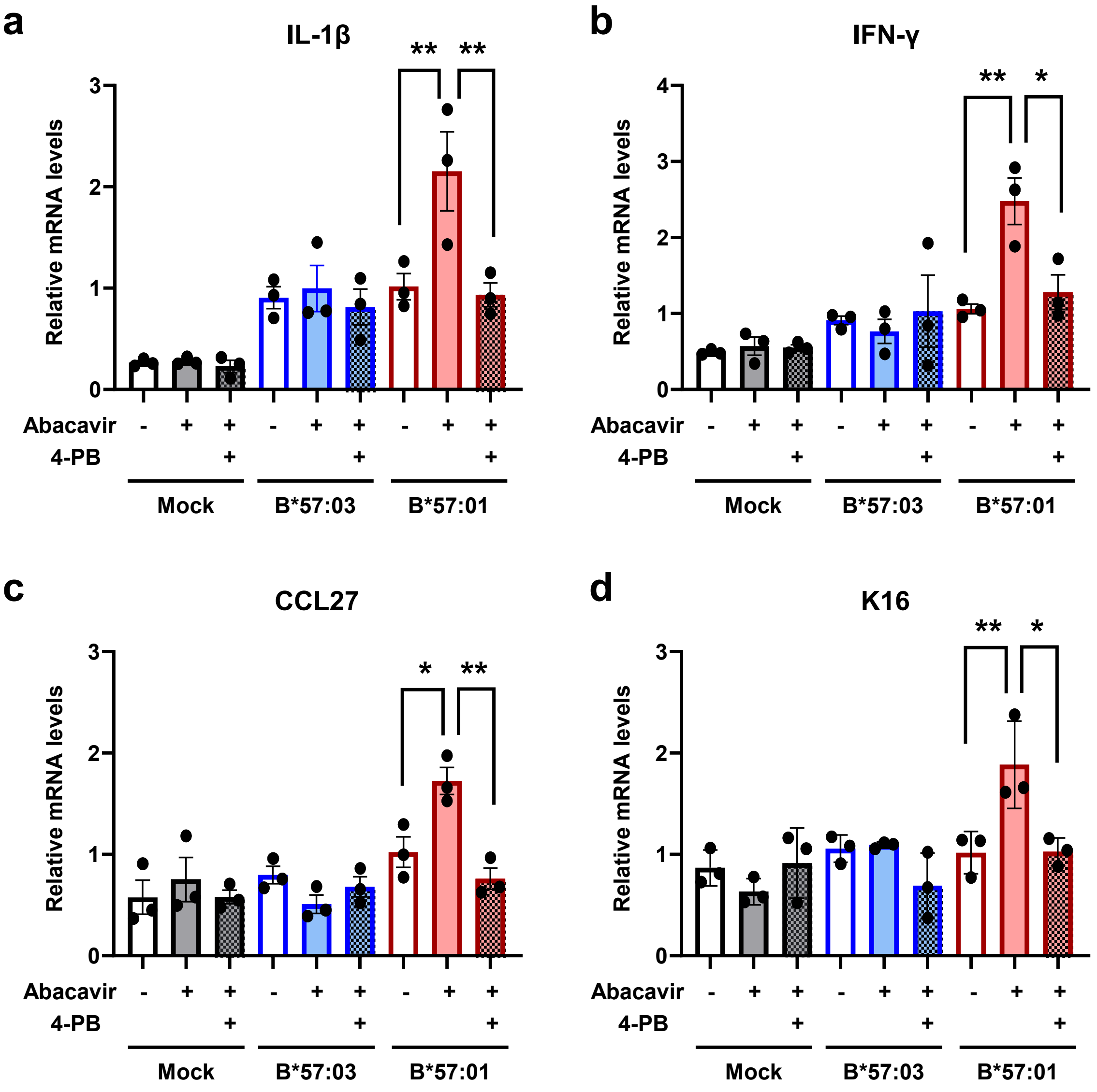

Fig. S5. Effects of abacavir on mRNA expression levels of cytokines and chemokines in HLA-transfected HaCaT cells. **a-d** mRNA expression levels of IL-1β (**a**), IFN-γ (**b**), CCL27 (**c**), and K16 (**d**) relative to GAPDH in HLA-B*57:01-, B*57:03-, or mock mRNA-transfected HaCaT cells. After 4-phenylbutyrate (4-PB) pretreatment, HaCaT cells were incubated in the presence (+)/absence (-) of 100 μM abacavir for 12 h. mRNA levels are presented in relation to the mean value in B*57:01 mRNA-transfected HaCaT cells without abacavir exposure. Data are expressed as the mean ± S.E.M. (n = 3/group). There were significant differences (* $p < 0.05$, ** $p < 0.01$) compared with another group (one-way ANOVA, followed by Bonferroni's multiple comparisons tests).

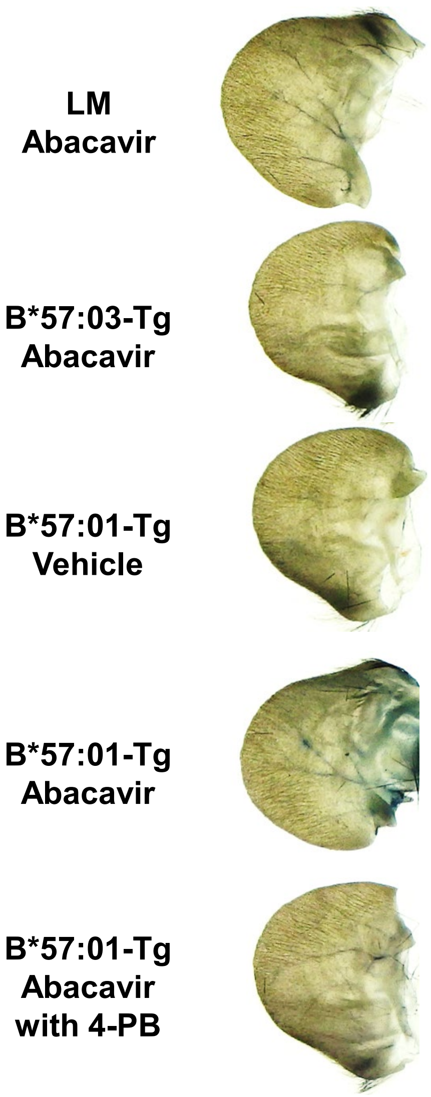

Fig. S6. Evans blue dye permeability assay for the investigation of vascular permeability in auricle skin tissue in abacavir administered B*57:01-Tg, their littermates (LM) and B*57:0-Tg. 6 h before 1% Evans blue injection (200 μL *i.v.*), B*57:01-Tg, LM, B*57:03-Tg with/without 4-phenylbutyrate (4-PB) pretreated were treated with 30 mg/body abacavir *p.o.* 30 min after 1% Evans blue injection, auricular tissue was removed and photographed. Images are representative of 4–12 independent experiments.

Vascular permeability in auricle

5.5 h after a single dose of 30 mg/body abacavir or vehicle *p.o.,* mice received an intravenous injection of 1% Evans blue. 30 min after the intravenous injection of Evans blue, the cervical spine of the mice was dislocated and the auricle was immediately collected and photographed.

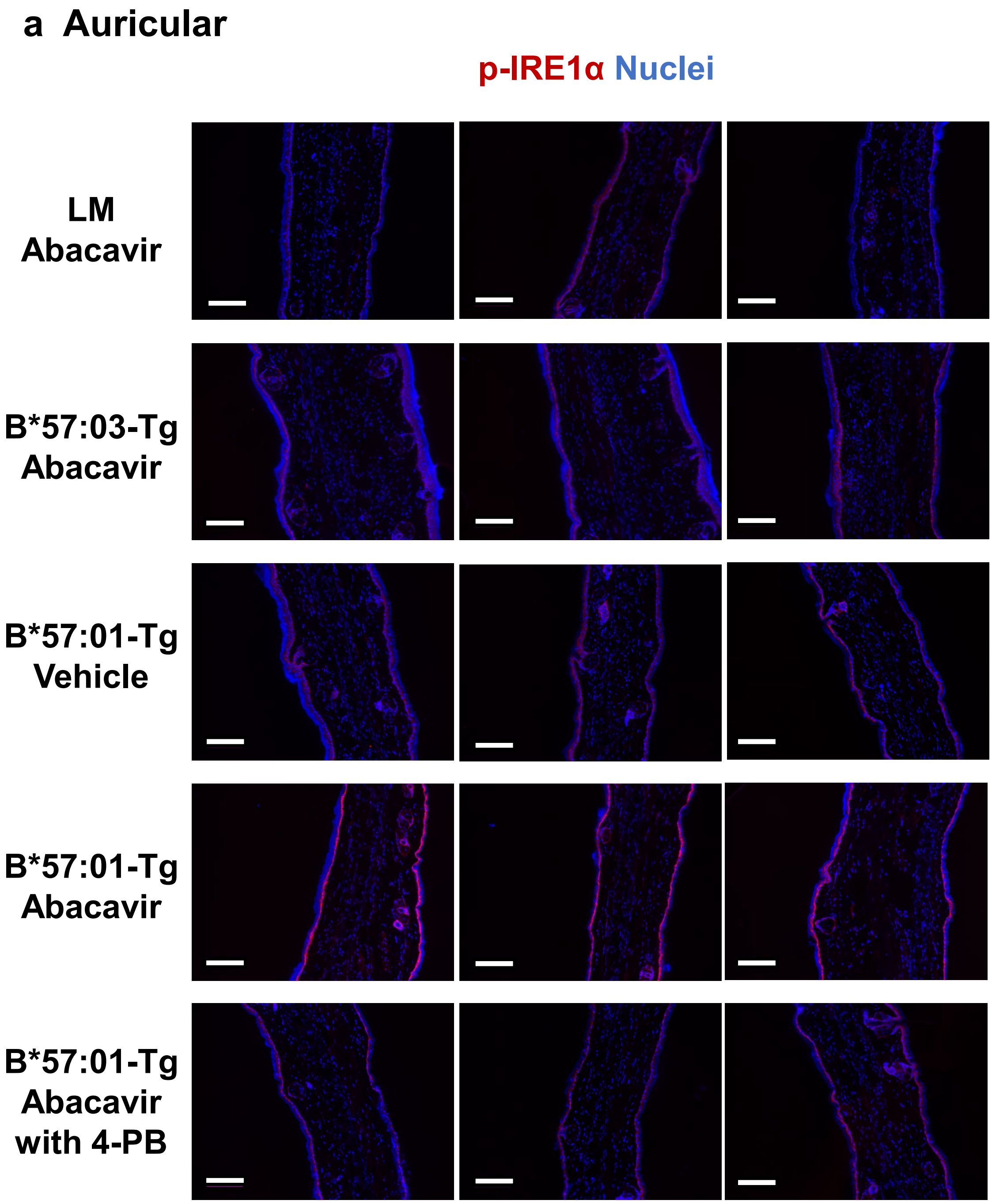
a Auricular
p-IRE1α Nuclei
LM
Abacavir
B*57:03-Tg
Abacavir
B*57:01-Tg
Vehicle
B*57:01-Tg
Abacavir
B*57:01-Tg
Abacavir
with 4-PB

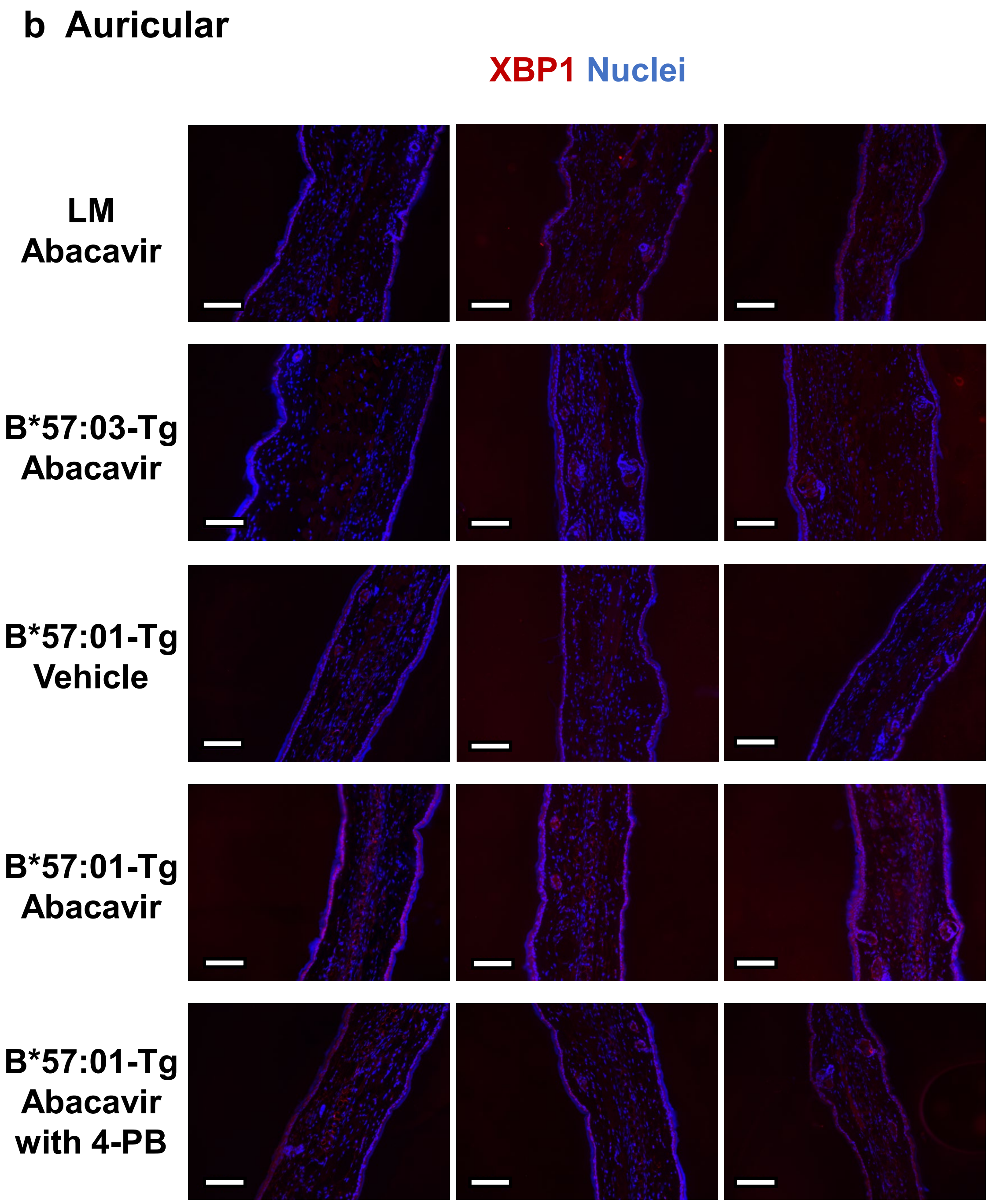
b Auricular
XBP1 Nuclei
LM
Abacavir
B*57:03-Tg
Abacavir
B*57:01-Tg
Vehicle
B*57:01-Tg
Abacavir
B*57:01-Tg
Abacavir
with 4-PB

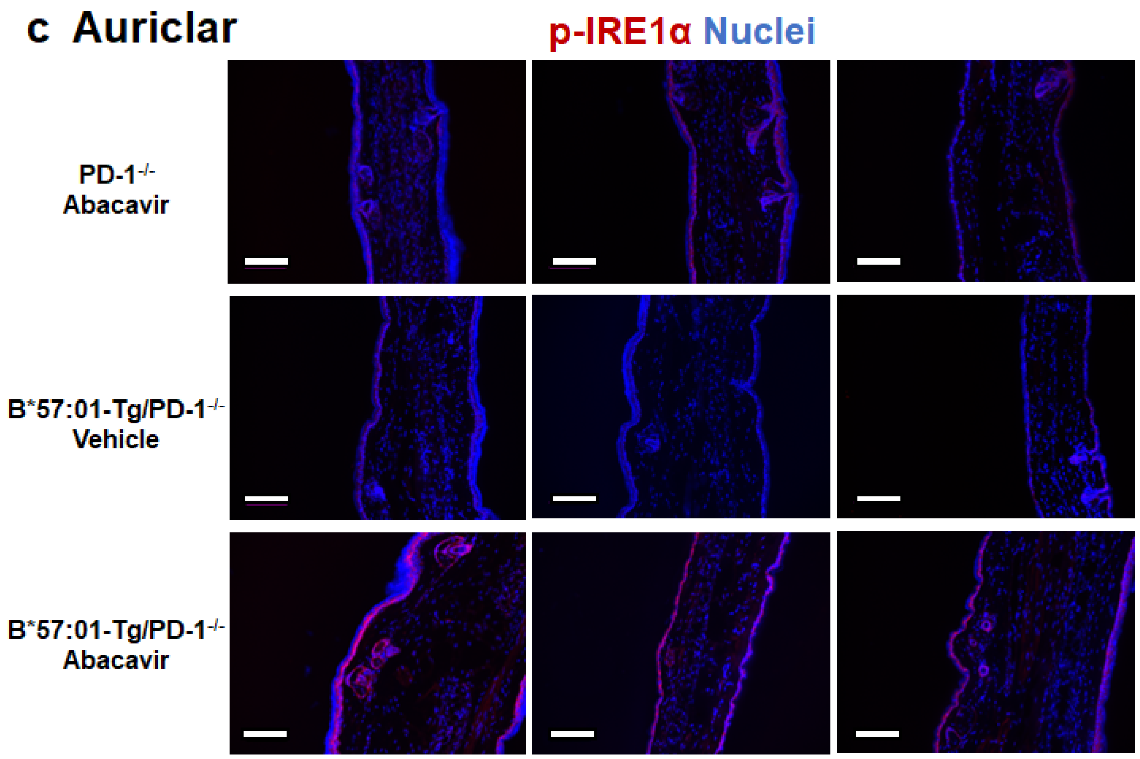
c Auriclar
p-IRE1α Nuclei
PD-1-/-
Abacavir
B*57:01-Tg/PD-1-/-
Vehicle
B*57:01-Tg/PD-1-/-
Abacavir

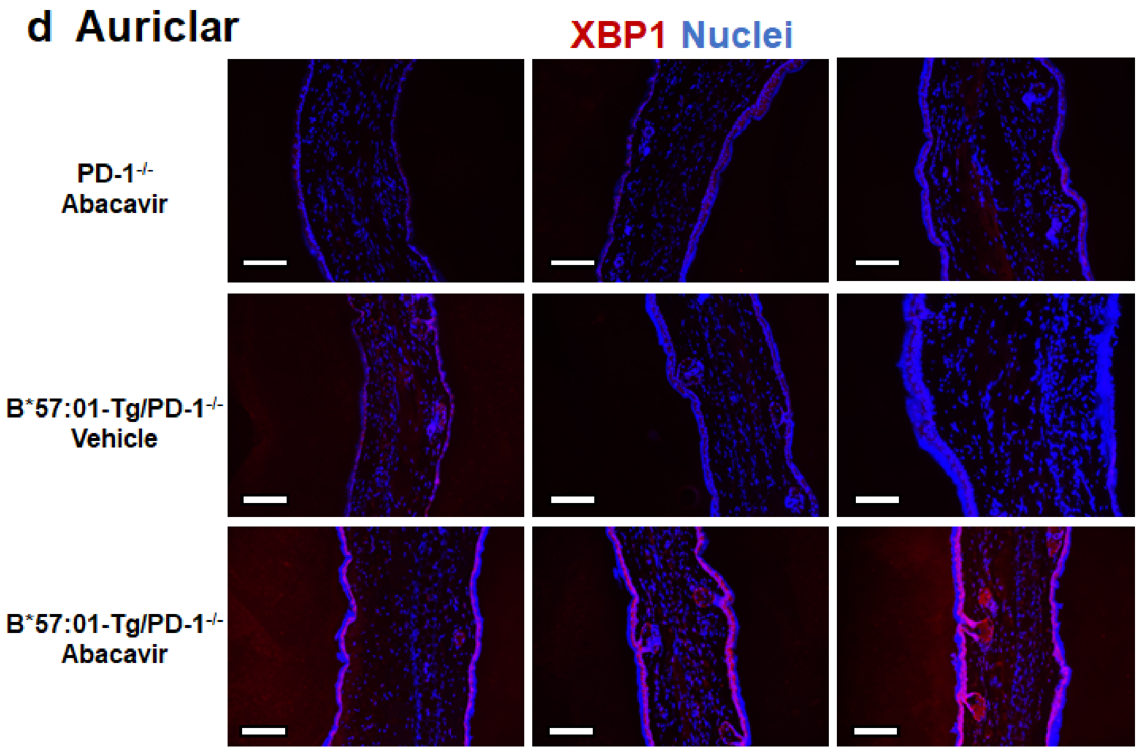
d Auriclar
XBP1 Nuclei
PD-1-/-
Abacavir
B*57:01-Tg/PD-1-/-
Vehicle
B*57:01-Tg/PD-1-/-
Abacavir

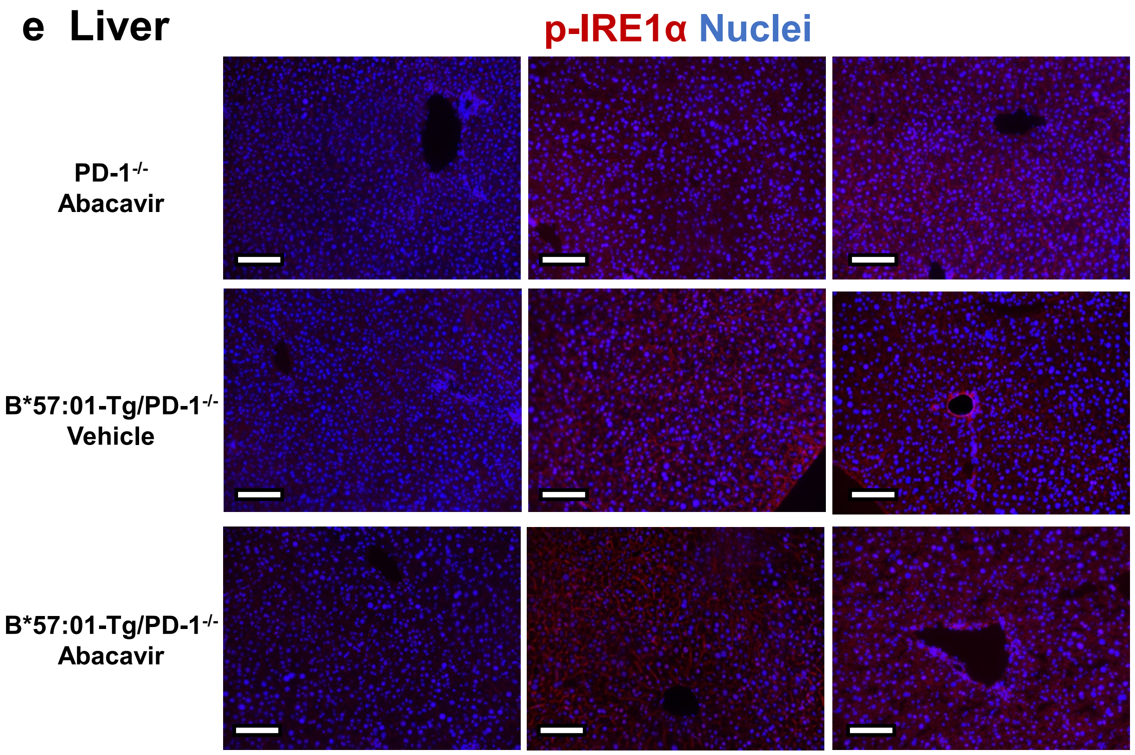
e Liver
p-IRE1α Nuclei
PD-1-/-
Abacavir
B*57:01-Tg/PD-1-/-
Vehicle
B*57:01-Tg/PD-1-/-
Abacavir

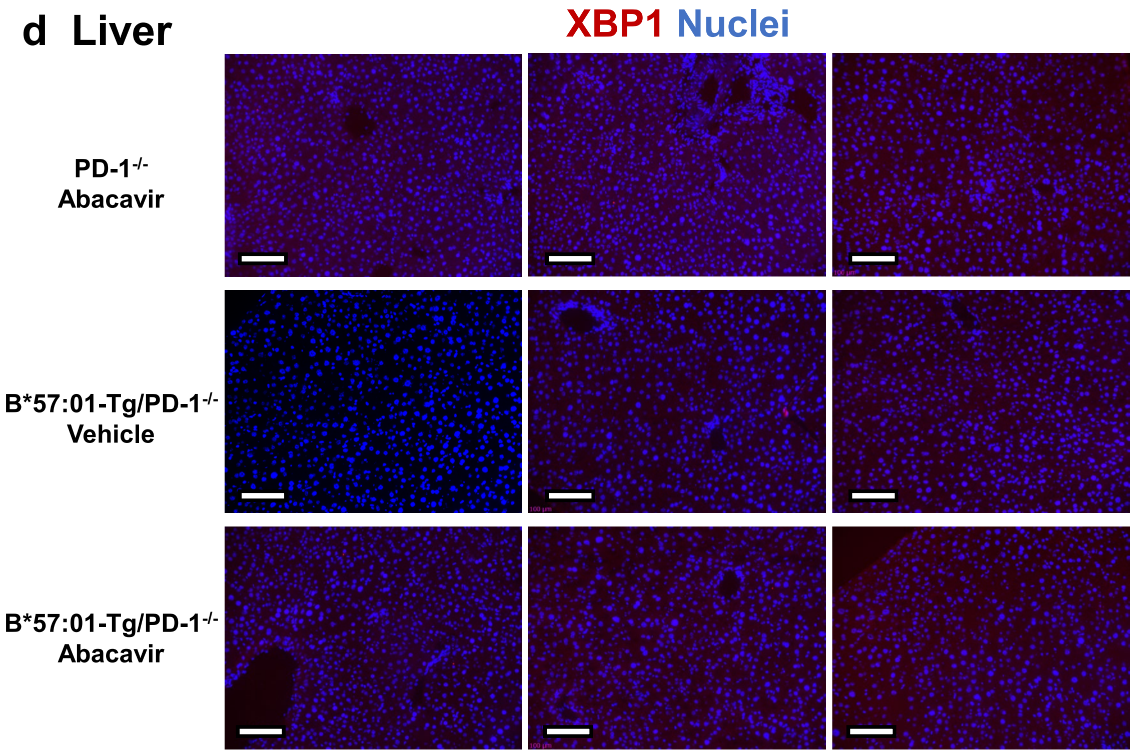
d Liver
XBP1 Nuclei
PD-1-/-
Abacavir
B*57:01-Tg/PD-1-/-
Vehicle
B*57:01-Tg/PD-1-/-
Abacavir

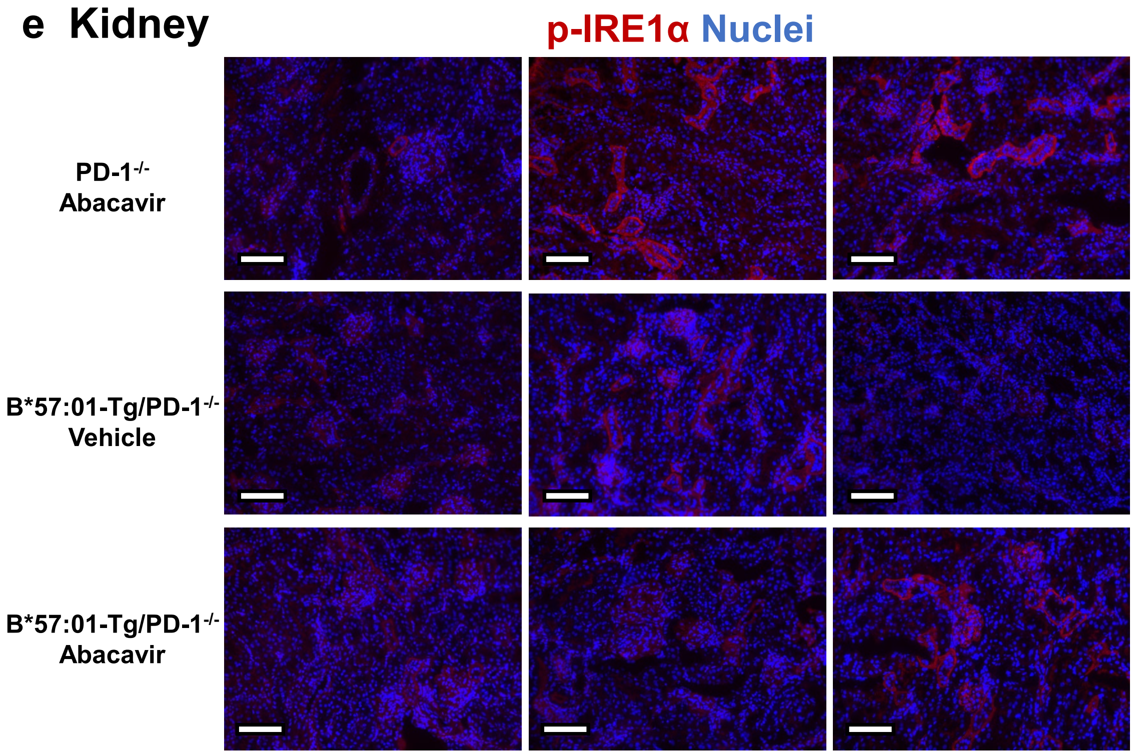
e Kidney
p-IRE1α Nuclei
PD-1-/-
Abacavir
B*57:01-Tg/PD-1-/-
Vehicle
B*57:01-Tg/PD-1-/-
Abacavir

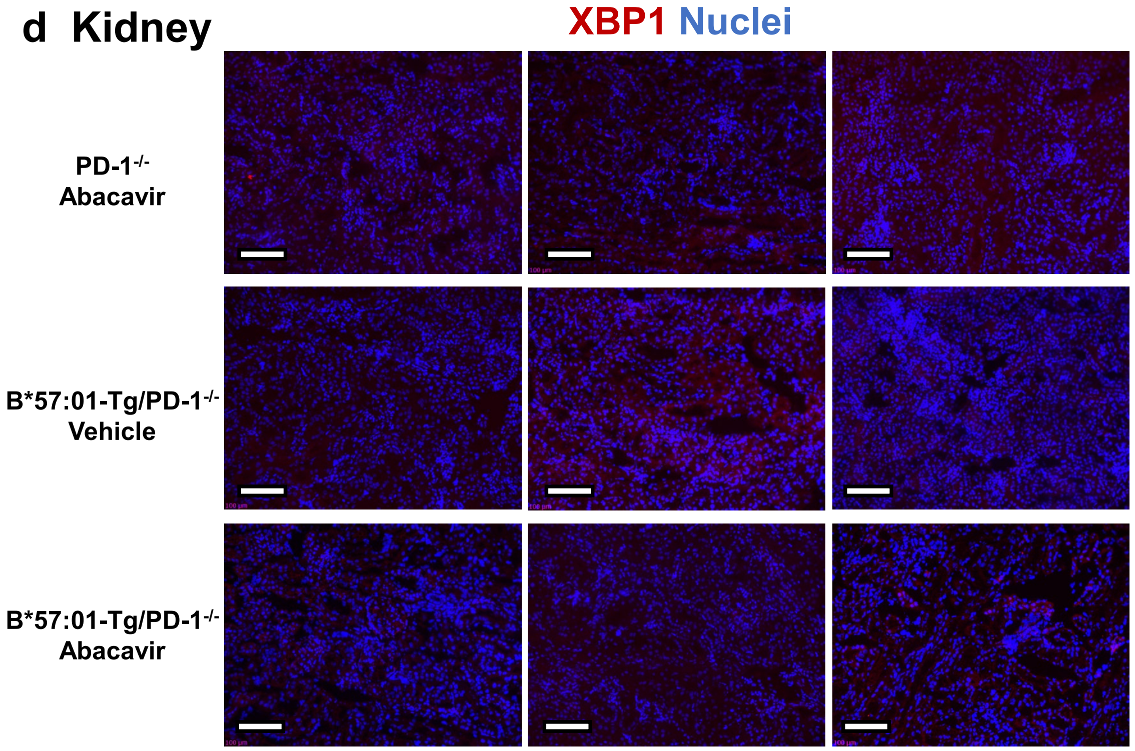
d Kidney
XBP1 Nuclei
PD-1-/-
Abacavir
B*57:01-Tg/PD-1-/-
Vehicle
B*57:01-Tg/PD-1-/-
Abacavir

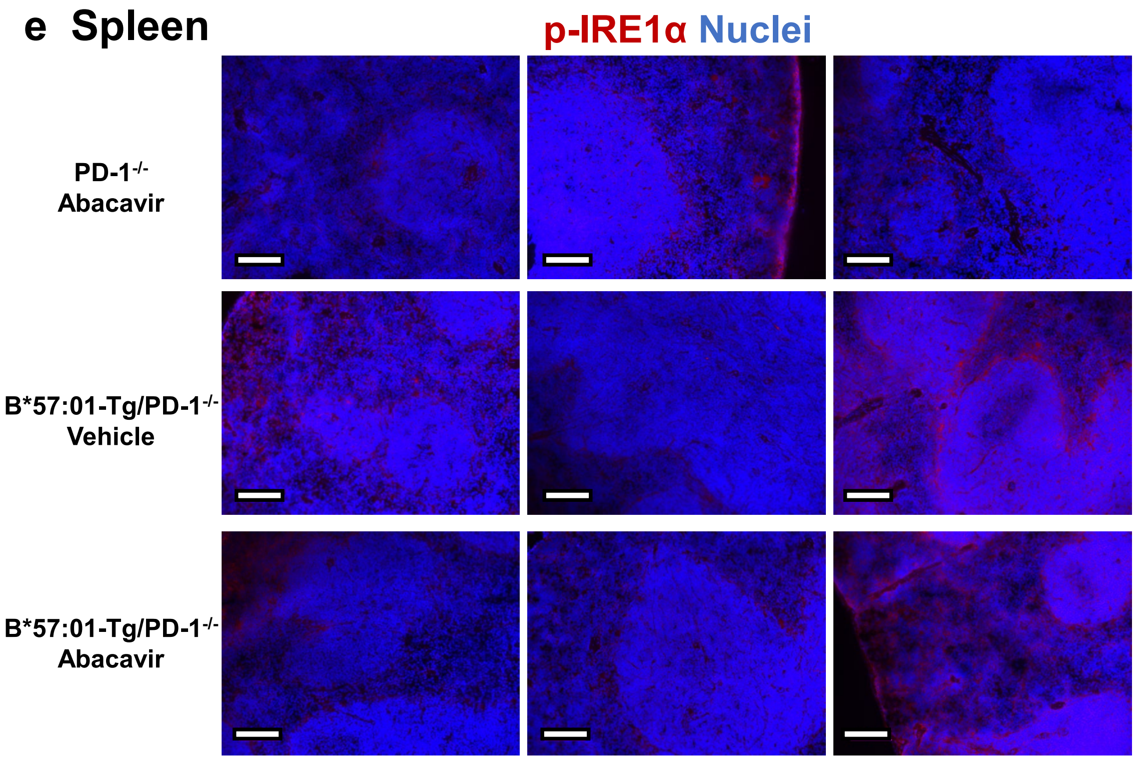
e Spleen
p-IRE1α Nuclei
PD-1-/-
Abacavir
B*57:01-Tg/PD-1-/-
Vehicle
B*57:01-Tg/PD-1-/-
Abacavir

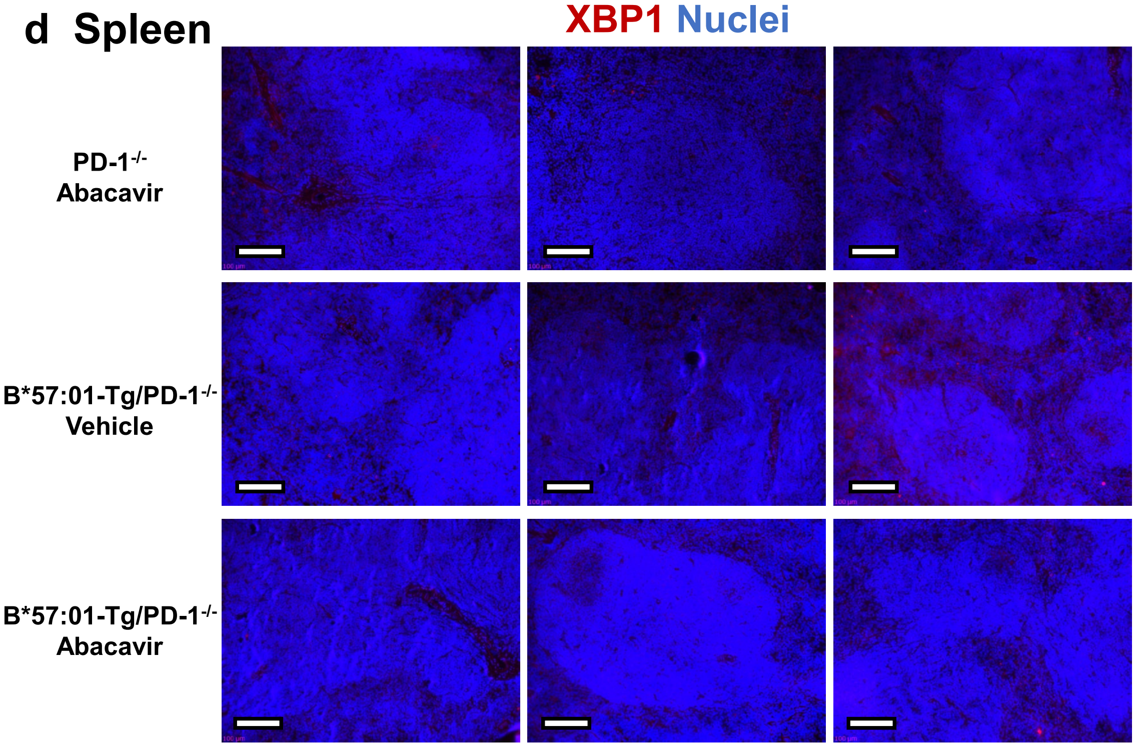
d Spleen
XBP1 Nuclei
PD-1-/-
Abacavir
B*57:01-Tg/PD-1-/-
Vehicle
B*57:01-Tg/PD-1-/-
Abacavir

Fig. S7. ER stress response in several tissues of B*57:01-Tg caused by abacavir. **a, b** Representative images of the auricular sections stained with anti-phosphorylated IRE1α (p-IRE1α; **a**) or XBP1 (**b**) antibody (red) and Hoechst 33342 (blue; nucleus staining) in B*57:01-Tg, their littermates (LM), and B*57:03-Tg. 3 h before auricular tissue removal, mice with/without 4-phenylbutyrate (4-PB) treatment were treated with 20 mg/body abacavir p.o. Each scale bar represents 100 µm. **c-j** Representative images of the auricular (**c, d**), liver (**e, f**), kidney (**g, h**), and spleen (**i, j**) sections stained with anti- p-IRE1α (**c, e, g, i**) or XBP1 (**d, f, h, j**) antibody (red) and Hoechst 33342 (blue; nucleus staining) in PD-1-deficient B*57:01-Tg (B*57:01-Tg/PD-1$^{-/-}$) and their littermates (PD-1$^{-/-}$). 3 h before auricular tissue removal, mice with/without 4-phenylbutyrate (4-PB) treatment were treated with 20 mg/body abacavir p.o. Each scale bar represents 100 µm.

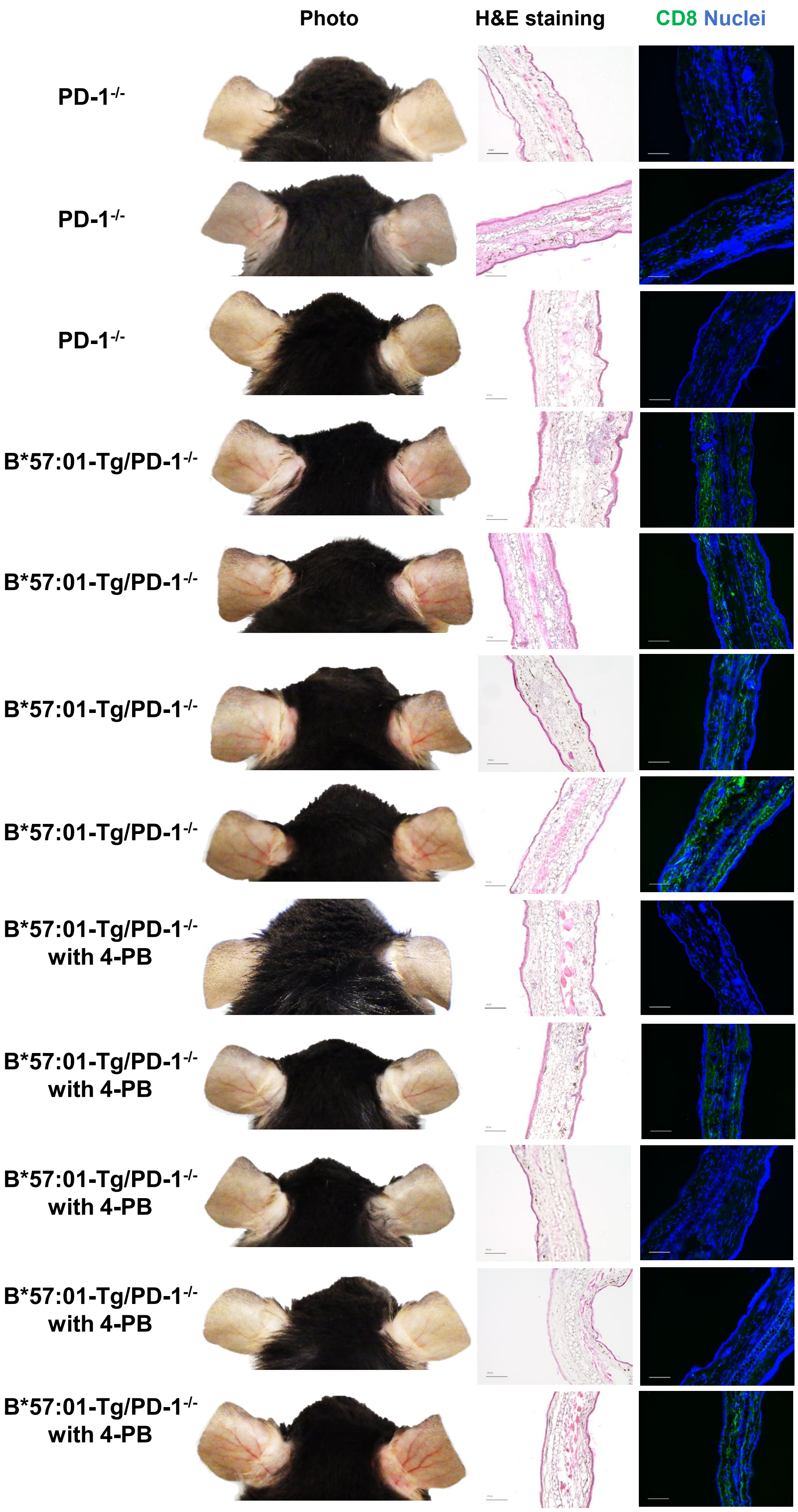
Photo
H&E staining
CD8 Nuclei
PD-1-/-
PD-1-/-
PD-1-/-
B*57:01-Tg/PD-1-/-
B*57:01-Tg/PD-1-/-
B*57:01-Tg/PD-1-/-
B*57:01-Tg/PD-1-/-
B*57:01-Tg/PD-1-/- with 4-PB
B*57:01-Tg/PD-1-/- with 4-PB
B*57:01-Tg/PD-1-/- with 4-PB
B*57:01-Tg/PD-1-/- with 4-PB
B*57:01-Tg/PD-1-/- with 4-PB

Fig. S8. Images of photos of the auricular or auricular section either stained with H&E or stained with anti-CD8 antibody (green) and Hoechst 33342 (blue; nucleus staining) in PD-1-deficient B*57:01-Tg (B*57:01-Tg/PD-$1^{-/-}$) and their littermates (PD-$1^{-/-}$). $CD4^+$ T cell-depleted B*57:01-Tg/PD-$1^{-/-}$ or PD-$1^{-/-}$, with/without 4-PB treatment, treated with 20 mg/body/day abacavir *p.o.* for 5 days.

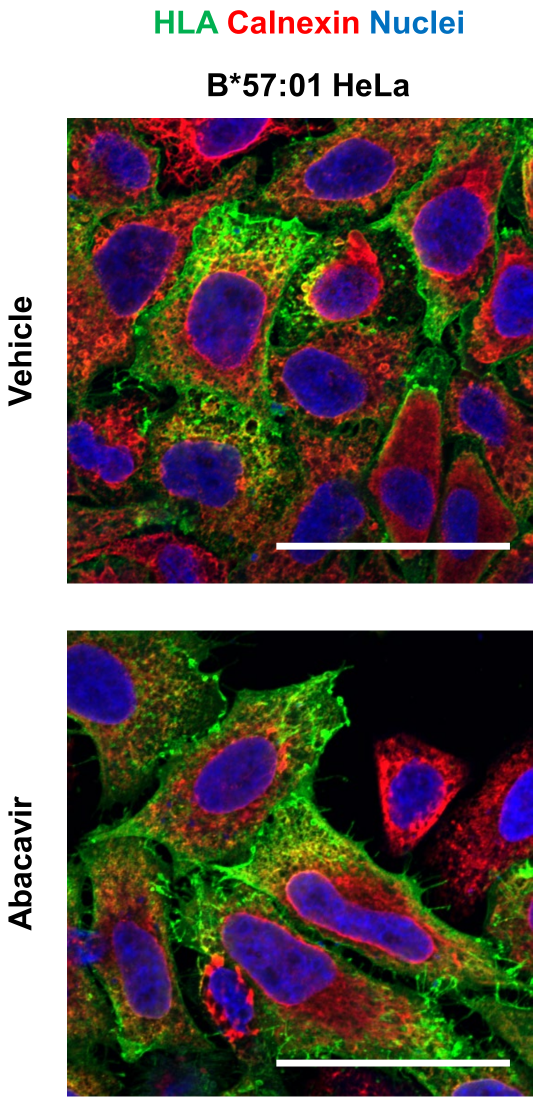

Fig. S9. Localization of FLAG-tagged HLA-B*57:01 in B*57:01-transfected HeLa cells. B*57:01-transfected HeLa cells were incubated in the presence or absence of 100 μM abacavir for 30 min. The cells were stained with anti-FLAG antibody (green), anti-calnexin antibody (red), and TO-PRO®-3 (blue; nucleus staining). Each scale bar represents 50 μm.

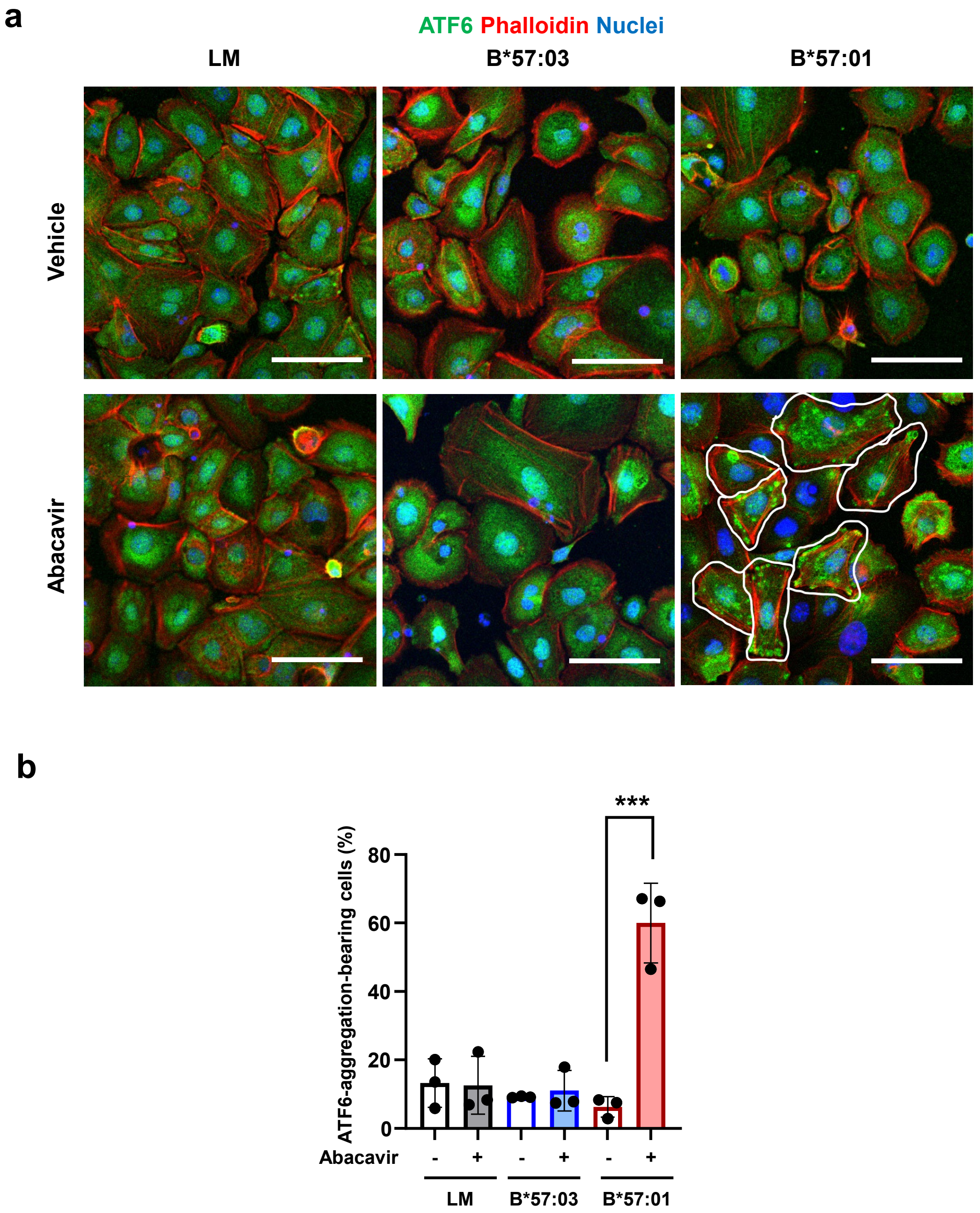

Fig. S10. Effects of abacavir on ATF6 intracellular localization in keratinocytes of HLA-Tg and LM. **a, b** Keratinocytes were incubated with (+)/without (-) 100 μM abacavir for 30 min. Keratinocytes were stained with anti-ATF6 antibody (green), rhodamine phalloidin (red; actin staining), and TO-PRO®-3 (blue; nucleus staining). 186–272 cells were observed for each experiment, and ATF6-aggregation-bearing cells were determined as indicated by the white line. Each scale bar represents 50 μm. Mean ± S.E.M. of three independent experiments is shown. There were significant differences (*** $p < 0.001$) compared with another group (one-way ANOVA, followed by Bonferroni's multiple comparisons tests).

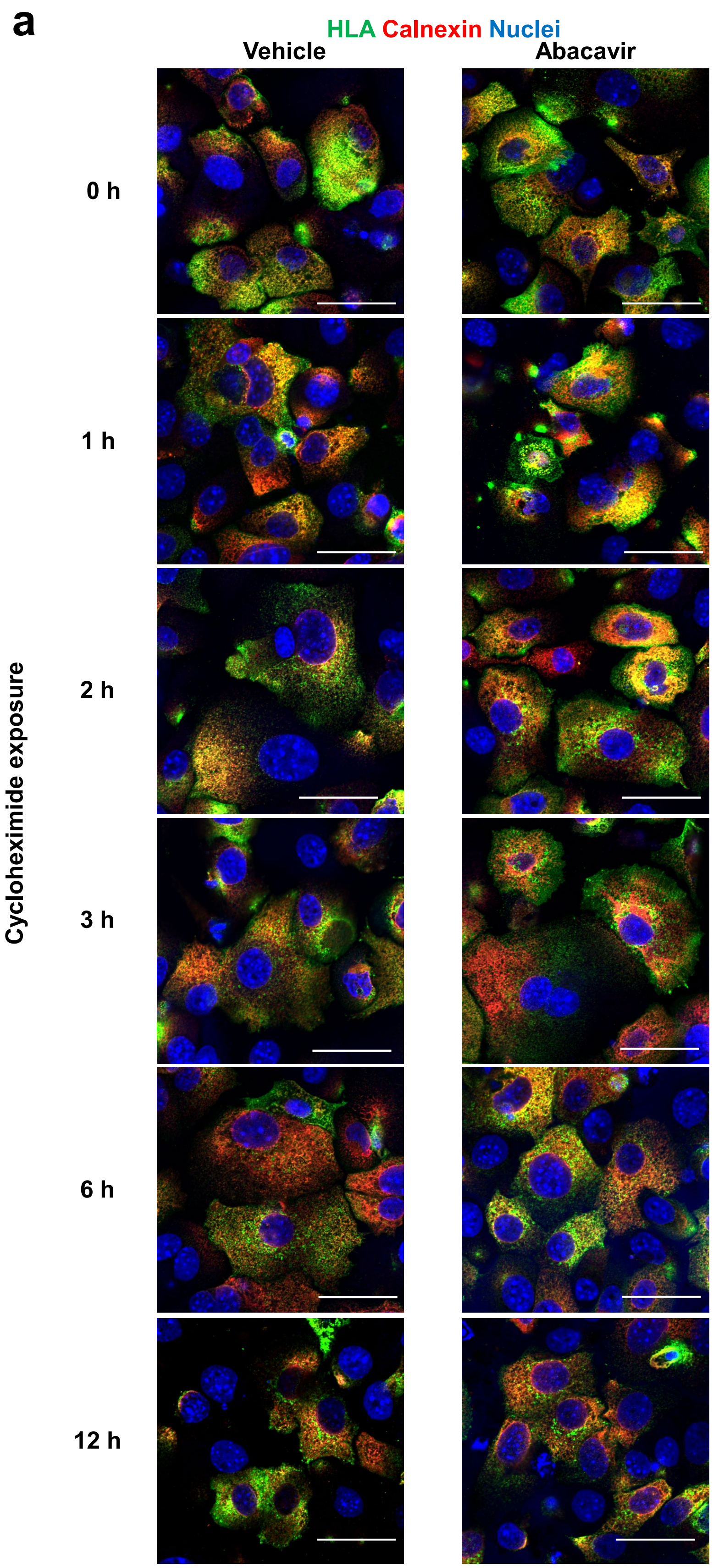
a
HLA Calnexin Nuclei
Vehicle
Abacavir
0 h
1 h
2 h
3 h
6 h
12 h
Cycloheximide exposure

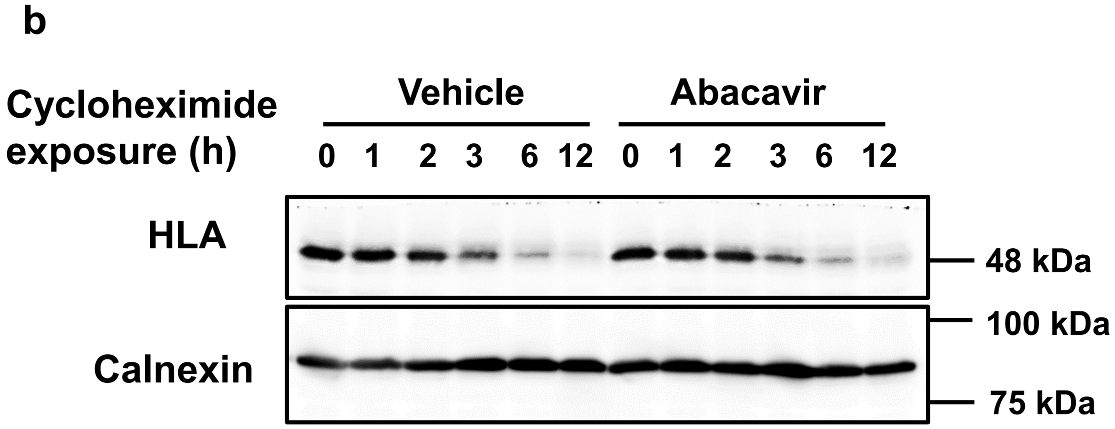

Fig. S11. Cycloheximide chase of HLA in keratinocytes from B*57:01-Tg (B*57:01-KCs). B*57:01-KCs were incubated in 20 μg/mL cycloheximide-contained medium with or without 100 μM abacavir for 0, 1, 2, 3, 6, 12 h. Panel **a** depicts changes in FLAG-tagged HLA intracellular localization in B*57:01-KCs following abacavir exposure. Cells were stained with anti-calnexin (red), anti-FLAG antibody (green) and TO-PRO®-3 (blue; for nucleus staining). Each scale bar represents 50 μm. Panel **b** presents the effects of abacavir on the degradation of HLA. FLAG-tagged HLA was detected by immunoblotting, with calnexin used as the loading control.

Cycloheximide chase of HLA in keratinocytes

20 μg/mL cycloheximide was dissolved in cultured medium and exposed to keratinocytes for 0, 1, 2, 3, 6, 12 h. 100 μM abacavir was exposed concurrently with cycloheximide. After that, cells were lysed or fixed. Whole cell lysate was used for HLA measurement by immunoblotting. Fixed cells were used to investigate the localization of HLA by immunocytochemistry.

Table S1. The list of gene sets which were significantly higher values (NOM p < 0.05) in B*57:01-KCs than B*57:03-KCs under abacavir exposure, resulting in GO analysis of microarray data.

	Gene Set	NES	NOM p-val
1	GOMF_CALCIUM_ION_TRANSMEMBRANE_TRANSPORTER_ACTIVITY	2.12	0.003
2	GOBP_STRIATED_MUSCLE_CONTRACTION	2.08	0
3	GOCC_SYNAPSE	2.06	0.001
4	GOBP_CARDIAC_MUSCLE_CONTRACTION	1.98	0.003
5	HP_BRUISING_SUSCEPTIBILITY	1.93	0.008
6	GOBP_SPHINGOLIPID_BIOSYNTHETIC_PROCESS	1.92	0.006
7	HP_ABNORMAL_CEREBRAL_ARTERY_MORPHOLOGY	1.92	0.009
8	GOBP_NEGATIVE_REGULATION_OF_TRANSPORT	1.87	0.008
9	GOBP_SECOND_MESSENGER_MEDIATED_SIGNALING	1.85	0.019
10	GOBP_MUSCLE_SYSTEM_PROCESS	1.85	0.006
11	GOBP_AMIDE_BIOSYNTHETIC_PROCESS	1.84	0.007
12	GOBP_RESPONSE_TO_ENDOPLASMIC_RETICULUM_STRESS	1.83	0.007
13	GOBP_CYCLIC_NUCLEOTIDE_MEDIATED_SIGNALING	1.82	0.01
14	GOCC_NEURON_TO_NEURON_SYNAPSE	1.8	0.007
15	GOBP_CALCIUM_ION_HOMEOSTASIS	1.8	0.018
16	HP_ABNORMAL_SCLERA_MORPHOLOGY	1.79	0.012
17	GOBP_SUPRAMOLECULAR_FIBER_ORGANIZATION	1.78	0.007
18	GOBP_PROTEIN_LOCALIZATION_TO_PLASMA_MEMBRANE	1.78	0.016
19	HP_NEOPLASM_OF_THE_SKIN	1.78	0.016
20	GOBP_MONOATOMIC_ION_HOMEOSTASIS	1.78	0.008
21	GOCC_POSTSYNAPTIC_SPECIALIZATION_MEMBRANE	1.78	0.014
22	GOBP_GLYCOSYLATION	1.77	0.019
23	HP_ABNORMAL_ADIPOSE_TISSUE_MORPHOLOGY	1.77	0.011

24	GOBP_SYNAPTIC_SIGNALING	1.75	0.008
25	GOBP_MUSCLE_CONTRACTION	1.75	0.021
26	GOBP_DETECTION_OF_STIMULUS	1.75	0.01
27	GOBP_INORGANIC_ION_HOMEOSTASIS	1.73	0.017
28	GOCC_RIBONUCLEOPROTEIN_COMPLEX	1.73	0.024
29	GOBP_CENTRAL_NERVOUS_SYSTEM_NEURON_DIFFERENTIATION	1.73	0.026
30	GOBP_PROTEIN_LOCALIZATION_TO_CELL_PERIPHERY	1.72	0.02
31	GOBP_NEGATIVE_REGULATION_OF_PROTEIN_LOCALIZATION	1.72	0.012
32	GOMF_ODORANT_BINDING	1.72	0.022
33	GOBP_PEPTIDE_BIOSYNTHETIC_PROCESS	1.71	0.024
34	GOCC_CORTICAL_CYTOSKELETON	1.71	0.022
35	GOBP_FOCAL_ADHESION_ASSEMBLY	1.7	0.025
36	HP_CHEST_PAIN	1.7	0.03
37	HP_SUBCUTANEOUS_HEMORRHAGE	1.7	0.019
38	GOCC_RECEPTOR_COMPLEX	1.7	0.014
39	GOCC_PLASMA_MEMBRANE_SIGNALING_RECEPTOR_COMPLEX	1.68	0.039
40	GOBP_CARBOHYDRATE_BIOSYNTHETIC_PROCESS	1.68	0.032
41	HP_NEOPLASM_OF_THE_ENDOCRINE_SYSTEM	1.68	0.027
42	GOBP_CELL_CELL_ADHESION_VIA_PLASMA_MEMBRANE_ADHESION_MOLECULES	1.68	0.029
43	GOBP_REGULATION_OF_CALCIUM_MEDIATED_SIGNALING	1.67	0.031
44	GOBP_CELL_JUNCTION_ORGANIZATION	1.66	0.029
45	GOMF_CARBOHYDRATE_BINDING	1.66	0.03
46	GOBP_DETECTION_OF_ABIOTIC_STIMULUS	1.65	0.028
47	GOMF_SERINE_HYDROLASE_ACTIVITY	1.65	0.036

48	GOBP_PROTEIN_MODIFICATION_BY_SMALL_PROTEIN_CONJUGATION	1.65	0.026
49	GOBP_REGULATION_OF_MUSCLE_SYSTEM_PROCESS	1.65	0.02
50	HP_SARCOMA	1.64	0.033
51	GOBP_POSITIVE_REGULATION_OF_CHEMOTAXIS	1.64	0.035
52	GOBP_SENSORY_PERCEPTION_OF_SMELL	1.64	0.036
53	GOMF_CALCIUM_ION_BINDING	1.64	0.017
54	GOBP_REGULATION_OF_PHOSPHOLIPASE_C_ACTIVITY	1.64	0.026
55	GOCC_POSTSYNAPTIC_DENSITY_MEMBRANE	1.63	0.031
56	GOBP_BEHAVIOR	1.63	0.031
57	GOMF_MONOATOMIC_CATION_TRANSMEMBRANE_TRANSPORTER_ACTIVITY	1.63	0.032
58	GOBP_REGULATION_OF_HEART_CONTRACTION	1.62	0.044
59	GOBP_RESPONSE_TO_NITROGEN_COMPOUND	1.61	0.019
60	GOBP_CALCIUM_ION_TRANSPORT	1.61	0.032
61	GOBP_PROTEASOMAL_PROTEIN_CATABOLIC_PROCESS	1.61	0.04
62	GOBP_NEGATIVE_REGULATION_OF_ESTABLISHMENT_OF_PROTEIN_LOCALIZATION	1.61	0.041
63	GOBP_CELLULAR_RESPONSE_TO_OXIDATIVE_STRESS	1.6	0.044
64	GOMF_MONOATOMIC_ION_TRANSMEMBRANE_TRANSPORTER_ACTIVITY	1.59	0.042
65	GOBP_CAMP_MEDIATED_SIGNALING	1.59	0.041
66	GOBP_REGULATION_OF_RNA_SPLICING	1.59	0.048
67	GOCC_GLUTAMATERGIC_SYNAPSE	1.59	0.049
68	GOBP_CELLULAR_RESPONSE_TO_CHEMICAL_STRESS	1.58	0.049
69	GOBP_HEART_PROCESS	1.58	0.039
70	GOBP_REGULATION_OF_TRANS_SYNAPTIC_SIGNALING	1.58	0.039
71	GOBP_CELL_SUBSTRATE_JUNCTION_ORGANIZATION	1.58	0.049

72	GOBP_REGULATION_OF_AMIDE_METABOLIC_PROCESS	1.58	0.037
73	GOCC_DENDRITIC_TREE	1.58	0.039
74	GOBP_REGULATION_OF_PHOSPHOLIPASE_ACTIVITY	1.57	0.045
75	GOCC_NUCLEAR_OUTER_MEMBRANE_ENDOPLASMIC_RETICULUM_MEMBRANE_NETWORK	1.57	0.037
76	GOMF_CIS_REGULATORY_REGION_SEQUENCE_SPECIFIC_DNA_BINDING	1.55	0.04
77	GOBP_CIRCULATORY_SYSTEM_PROCESS	1.54	0.045
78	GOBP_DIGESTIVE_SYSTEM_DEVELOPMENT	1.53	0.048
79	GOCC_POSTSYNAPTIC_SPECIALIZATION	1.53	0.043
80	GOMF_DNA_BINDING_TRANSCRIPTION_FACTOR_ACTIVITY	1.53	0.044

GOBP: GO biological process, GOCC: GO cellular component, GOMF: GO molecular function, HP: Human Phenotype.

Table S2. Primers used for real-time PCR assay.

Gene	Forward primer (5′→3′)	Reverse primer (5′→3′)
Mouse Xbp1s	AAGAACACGCTTGGGAATGG	CTGCACCTGCTGCGGAC
Mouse Chop	CCCAGGAAACGAAGAGGAAG	AGTGCAGTGCAGGGTCACAT
Mouse Bip	ACTTGGGGACCACCTATTCCT	ATCGCCAATCAGACGCTCC
Mouse Pdgfra	CAACCACACTCAGACGGATG	GCGGCAAGGTATGATGCCAGAG
Mouse Itpr1	AGACCTCTGCCTTAGGA GGTATTT	ACTGGGCAGGCATATATAGTTAGC
Mouse Ube2j1	GCTCAGCCTCTGGAGGATAA	CCGTGATAAACTCCTCCATCAA
Mouse Tmem129	CTGGAGTGCAGAATCGTTCA	GAGAGAAGGGGAAGGGTCAC
Mouse Hspa1a	ATATGTGGCCTTGAGGACTGTC	AAAGCCCACGTGCAATACAC
Mouse Hspa1l	ATCAATGAAGCCGGCAAACC	ACAGCGTTGGTGACATTGTG
Mouse Il1b	CAGGGACAGGATATGGAGCAAC	ACGCAGGACAGGTACAGATTC
Mouse Ifng	TGTGGAGACCATCAAGGAAGAC	TGCTTTGCGTTGGACATTCAAG
Mouse K16	ATGACCGCCTGGCCACCTACCTGGAC	CCCTCCACGGACTGCCGCAAGAA
Mouse Ccl27	CTTGCCTCTGCCCTCCAGCACTA	CCCTCCACGGACTGCCGCAAGAA
Mouse Gapdh	TGAAGTCGCAGGAGACAACC	ATGTGTCCGTCGTGGATCTG
Human IL-1β	CAGGGACAGGATATGGAGCAAC	CACGCAGGACAGGTACAGATTC
Human IFN-γ	TGTGGAGACCATCAAGGAAGAC	TGCTTTGCGTTGGACATTCAAG
Human K16	TGGAAGTGAAGATCCGTGACTGG	AAAATGGGCTGCGCATTCTCAA
Human CCL27	CTCAGCTCTACCGAAAGCC	GCCCATTTTCCTTAGCATCC
Human GAPDH	AAGGTCATCCCTGAGCTGAA	TTCTAGACGGCAGGTCAGGT

Movie. S1. Live cell imaging of calcium release from the ER to the cytoplasm in abacavir-exposed keratinocytes of HLA-B*57:01-Tg. Calcium release from the ER to the cytoplasm in keratinocytes was observed using a calcium fluorescent probe (Cal-520® AM) and Zeiss LSM 780 confocal microscope. Keratinocytes were incubated with Cal-520® AM and Hoechst 33342 for 1 h, then incubated with flesh medium with 100 μM abacavir for 44 min.

SI References

1. Kawase A*, et al.* (2021) Protein Kinase N Family Negatively Regulates Constitutive Androstane Receptor-Mediated Transcriptional Induction of Cytochrome P450 2b10 in the Livers of Mice. *J Pharmacol Exp Ther* 379(1):53-63.
